# Tissue-engineered cardiac patches enriched with IGF1 modified mRNA alleviate myocardial infarction by enhancing cell survival and angiogenesis

**DOI:** 10.1016/j.mtbio.2025.102686

**Published:** 2025-12-18

**Authors:** Bingqian Yan, Xuefeng Ai, Huijing Wang, Yao Tan, Yiqi Gong, Li Yang, Ying Chen, Tingting Lu, Minglu Liu, Runjiao Luo, Kaixiang Li, Xin Tang, Wei Wang, Wei Fu

**Affiliations:** a Institute of Pediatric Translational Medicine, Shanghai Institute of Pediatric Congenital Heart Disease, Shanghai Children's Medical Center, Shanghai Jiao Tong University School of Medicine, Shanghai 200127, China; b Department of Pediatric Cardiothoracic Surgery, Shanghai Children's Medical Center, Shanghai Jiao Tong University School of Medicine, Shanghai 200127, China; c Department of Anesthesiology, Fudan University Shanghai Cancer Center, Department of Oncology, Shanghai Medical College, Fudan University, Shanghai 200032, China; d Department of Hainan Key Laboratory of Molecular Medicine for Women and Children's Health, Hainan Branch, Shanghai Children's Medical Center, Shanghai Jiao Tong University School of Medicine, Sanya 572000, China; e Basic Medical College of Bengbu Medical University, Bengbu 233000, China; f Shanghai Key Laboratory of Tissue Engineering, Shanghai 200011, China

**Keywords:** Tissue-engineered cardiac patch, Modified mRNA, Insulin-like growth factor 1, Cardiac regeneration, Myocardial infarction, Induced pluripotent stem cells

## Abstract

Tissue-engineered cardiac patches (TECPs), which combine cells with biomaterial scaffolds, hold great promise for myocardial repair and regeneration. However, their broader application remains limited by the low survival rate of transplanted cells. To boost the therapeutic efficacy of cardiac patches, genetic engineering and localized delivery of bioactive factors are essential for optimizing cellular function in vivo. In this study, nanofibrous membranes composed of polycaprolactone-co-l-lactide (PLCL) and gelatin at various ratios were fabricated using electrospinning technology. Among these, membranes containing 30 % gelatin displayed optimal properties, promoting the adhesion, survival, proliferation, and cardiomyocyte differentiation of induced pluripotent stem cell-derived cardiac progenitor cells (iPSC-CPCs). Following this, TECPs were constructed in vitro and transfected with modified mRNA (modRNA) encoding insulin-like growth factor 1 (IGF1). Further evaluations revealed that IGF1 modified mRNA (modIGF1)-enriched TECPs significantly reduced infarct size, enhanced the survival and proliferation of transplanted cells, promoted vascularization and facilitated cardiac functional recovery. The integration of modRNA technology with myocardial patches facilitates the controlled release of therapeutic proteins, thereby preserving cellular function and offering a promising approach to advancing cardiac tissue engineering.

## Graphical abstract

**Figure:**
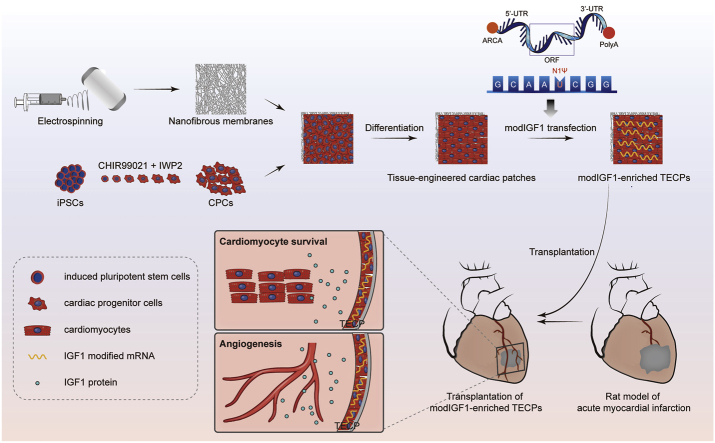

## 1 Introduction

Cardiovascular disease remains the leading cause of morbidity and mortality globally, imposing a significant burden on the public health [1,2]. Among these conditions, ischemic cardiomyopathy stands out as a major contributor [3]. The extensive necrosis of cardiomyocytes and compensatory proliferation of fibrotic tissue in ischemic regions lead to progressive ventricular remodeling and heart failure (HF). Despite substantial advancements in pharmacological and surgical treatments, these interventions have limited efficacy in regenerating or replacing damaged myocardium, resulting in an alarming 50 % 5-year survival rate for patients with HF even after treatment [4,5]. Heart transplantation, while considered the most viable therapeutic option, is constrained by donor scarcity and the risk of graft rejection. These challenges underscore the urgent need for the development of heart regenerative therapies [3,6]. Tissue-engineered cardiac patches (TECPs) not only provide mechanical support to the damaged myocardium but also mimic the extracellular microenvironment, which enhance cell survival and promote therapeutic benefits [[7], [8], [9]].

The selection and fabrication of scaffolds are pivotal to the advancement of myocardial tissue engineering. Natural materials, such as collagen [10] and gelatin [11,12], offer excellent biocompatibility and biodegradability. In contrast, synthetic polymers like poly(ε-caprolactone) (PCL) [13,14], poly(lactic acid) (PLA) [15], and poly(l-lactide-co-ε-caprolactone) (PLCL) [16,17] possess superior mechanical properties and can be extensively modified to match the biophysical and biochemical features of natural myocardium. Electrospun scaffolds that combine both synthetic and natural materials are particularly promising. These scaffolds, characterized by high porosity and structural similarity to the extracellular matrix, facilitate cell adhesion, diffusion and proliferation [8]. Moreover, they exhibit favorable mechanical properties and generate non-toxic degradation products, making them suitable for use in tissue-engineered myocardial patches [3].

Human induced pluripotent stem cells (hiPSCs) present a promising avenue for myocardial regeneration by offering large quantities of high-quality cardiac progenitor cells (CPCs) and cardiomyocytes (CMs). However, the low survival rate of transplanted cells, attributed to cell leakage and the ischemic-hypoxic environment at the infarct site, has prompted researchers to emphasize the delivery of critical bioactive factors through gene vectors [[18], [19], [20]]. Insulin-like growth factor 1 (IGF1) is a critical growth factor involved in the regulation of various physical processes in the heart. Numerous studies have shown that IGF1 inhibits apoptosis, promotes cell proliferation, enhances neovascularization, protects the heart from oxidative stress injury, and aids in the recovery of cardiac function following myocardial infarction [[21], [22], [23]]. However, prolonged and systemic administration of IGF1 can result in adverse effects, including an elevated risk of tumor development [24]. Modified mRNA (modRNA) has emerged as an innovative genetic vector that efficiently translates and expresses target proteins within a short time frame. This technology has been widely applied in various fields, including vaccine development [25,26], tumor immunotherapy [27,28], the treatment of cardiovascular [[29], [30], [31]] and genetic diseases [32,33]. Our previous studies combining modRNAs with cell therapy [34,35] and tissue-engineered constructs [36] have underscored the potential of modRNA technology in advancing cell therapy, tissue engineering, and regenerative medicine. Intriguingly, the early period following acute myocardial infarction is a critical timeframe, characterized by cardiomyocyte necrosis and apoptosis, inflammatory response, fibroblast infiltration and neovascularization, which aligns with the expression kinetics of modRNA [37]. Therefore, the use of IGF1 modified mRNA (modIGF1) might optimize therapeutic benefits while mitigating the potential risks, highlighting the advantages of modRNA as a gene delivery system in cardiac regenerative therapy.

**Fig. 1:**
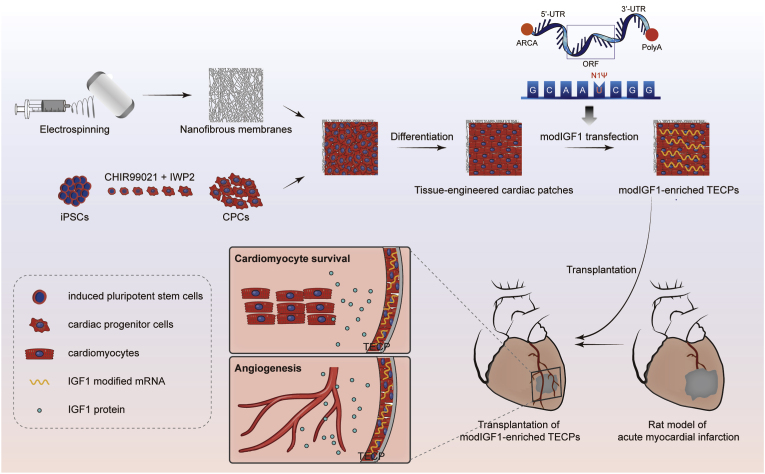
Graphical abstract illustrating therapeutic transplantation using modIGF1-enriched tissue-engineered cardiac patches in a rat model of myocardial infarction (MI).

Therefore, this study introduces a novel therapeutic strategy that leverages modRNA technology to engineer genetically enhanced cardiac patches capable of autocrine secretion of key bioactive factors (Fig. 1). Specifically, we generated TECPs by combining hiPSC-derived CPCs with PLCL/gelatin composite nanofibrous membranes and subsequently transfecting them with modIGF1. The innovation of this approach lies in the integration of modRNA-based genetic enhancement with hiPSC-derived cells within a biomaterial scaffold, enabling controlled and localized IGF1 delivery. To validate this strategy, we systematically evaluated the therapeutic efficacy of modIGF1-engineered TECPs in a rat model of acute myocardial infarction, focusing on their capacity to enhance cardiomyocyte survival and proliferation, promote neovascularization, and ultimately improve cardiac function.

## 2 Materials and methods

### 2.1 Fabrication of PLCL/gelatin electrospun nanofibers

PLCL (LA:CL = 50:50; Mn = 450,000, Daigang Biomateria, Jinan, China) and gelatin (48722-100G-F, Sigma-Aldrich) were dissolved in 1,1,1,3,3,3-hexafluoro-2-propanol (J&K Scientific, Beijing, China) at a concentration of 5 % (wt/v). Different mass ratios of PLCL to gelatin were prepared, specifically 100:0, 90:10, 70:30, 50:50, 30:70, and 0:100, corresponding to 0 %, 10 %, 30 %, 50 %, 70 %, and 100 % gelatin (Get) groups, respectively. The solutions were thoroughly mixed on a magnetic stirrer at room temperature. For electrospinning, the prepared solution was loaded into a 10 mL syringe with a 20-gauge blunt metal needle and mounted on a syringe pump (KDS100, KD Scientific, Holliston, MA, USA), with a feed rate of 1 mL/h. Electrospinning was conducted at a 12 cm working distance with an applied voltage of 10–12 kV using a high-voltage power supply (TXR1020N30-30, Teslaman, Dalian, China). To minimize environmental variations, electrospinning was performed under controlled conditions (temperature: 20–25 °C, humidity: 30–40 %). After fabrication, the electrospun materials were placed in a vacuum drying chamber for more than one week to ensure complete solvent removal.

### 2.2 Characterization of the nanofibrous scaffolds

The surface microstructure of the electrospun membranes was analyzed using a scanning electron microscope (SEM, TESCAN, Shanghai, China). For each group, 100 nanofibers were randomly selected from multiple SEM images to measure fiber diameters using Nano Measurer 1.2.5.

Mechanical properties were assessed using a tabletop uniaxial tensile testing machine (INSTRON-3343, Norwood, MA, USA). Rectangular nanofiber samples (30 mm × 10 mm × 0.15–0.20 mm) were subjected to uniaxial tensile testing at a constant strain rate of 10 mm/min. For each group, four dry samples and four hydrated samples (pre-soaked in PBS for 30 min at room temperature) were tested. Young's modulus was calculated from the linear elastic region of the stress-strain curve.

Water absorption capacity was evaluated as described in previous studies [38,39]. Briefly, four samples per group were weighed in the dry state (W_0_, g), incubated in double-distilled water at room temperature for 30 min, and reweighed after surface water removal to obtain the wet weight (W_w_, g). Water absorption was determined using the formula: Water absorption (%) = ((W_w_ – W_0_)/W_0_) × 100 (%).

Degradation rate was assessed as previously described [36]. Vacuum-dried nanofiber membranes were weighed (W_0_, g) and then immersed in PBS at a 37 °C incubator for 1, 2, 3, and 4 weeks. After each time point, those membranes were rinsed with deionized water, dried, and reweighed (W_w_, g). The degradation rate (%) was calculated using the formula: Degradation rate (%) = ((W_0_ – W_w_)/W_0_) × 100 (%).

Fourier Transform Infrared Spectroscopy (FTIR, Nicolet 460, Thermo Fisher Scientific, Waltham, MA, USA) was employed to analyze the chemical composition and functional groups of the electrospun nanofibrous membranes. Spectra were recorded using the KBr pellet technique over a scanning range of 500–4000 cm^−1^ with a resolution of 2 cm^−1^.

### 2.3 hiPSC culture and differentiation into CPCs and CMs

hiPSCs reprogrammed from human cord blood cells were obtained from Professor Yanxin Lee's group (Shanghai Children's Medical Center, School of Medicine, Shanghai Jiao Tong University). The maintenance and differentiation methods were consistent with our previous studies [[40], [41], [42]].

Briefly, hiPSCs were cultured on Matrigel (Corning)-coated 6-well plates and maintained in TeSR-E8 medium (STEMCELL) with daily medium changes. When cells reached 70–80 % confluence, they were detached using 0.5 mM EDTA (Invitrogen) and passaged onto new Matrigel-coated plates. To enhance cell survival, 5 μM Y-27632 (STEMCELL) was supplemented for the first 24 h. The cells were incubated at 37 °C with 5 % CO_2_.

Wnt/β-catenin signaling pathway was modulated to generate high-quality cardiac progenitor cells and cardiomyocytes from hiPSCs. On Day −2, hiPSCs were dissociated with Accutase (STEMCELL) and reseeded onto Matrigel-coated 12-well plates at a density of 1 × 10^6 cells/well in TeSR-E8 medium supplemented with 5 μM Y-27632. The medium was refreshed on Day −1. On Day 0, once the cells were fully confluent, TeSR-E8 medium was replaced with differentiation medium (RPMI 1640/B27 minus insulin medium (Gibco) supplemented with 12 μM GSK3 inhibitor CHIR99021 (STEMCELL)). After 30 h, the medium was replaced with RPMI 1640/B27 minus insulin medium. On Day 3, the medium was changed to a combined formulation (half conditioned medium and half fresh RPMI 1640/B27 minus insulin medium supplemented with 5 μM Wnt inhibitor IWP2 (STEMCELL)). On Day 5, the medium was switched back to RPMI 1640/B27 minus insulin. On Day 6, hiPSC-derived CPCs (hiPSC-CPCs) were harvested to construct TECPs. From Day 7 onward, RPMI 1640/B27 medium (Gibco) was used for cell maintenance, with medium changes occurring every two days. All cultures were maintained at 37 °C in a 5 % CO_2_ incubator.

### 2.4 Transmission electron microscopy

To examine the ultrastructural features of hiPSC-derived CMs (hiPSC-CMs), cell specimens were fixed in 2.5 % glutaraldehyde and 1 % osmium tetroxide for every 2 h at 4 °C. Subsequently, dehydration was carried out through a graded ethanol series for 10 min at each step and staining was performed with 3 % uranyl acetate in 70 % ethanol. Following this, samples were embedded in Epon 812, sectioned using an ultramicrotome (EM UC7, Leica, Wetzlar, Germany), and counterstained with lead citrate for imaging under a high-contrast transmission electron microscope (H-7650, Hitachi, Japan).

### 2.5 Electrophysiological characterization of hiPSC-CMs

hiPSC-CMs were plated on a glass-bottomed dish and positioned onto the stage of an inverted microscope (Ti–U, Nikon, Japan) with a patch clamp amplifier (MultiClamp700B, Axon CNS, USA). Patch electrodes (3–5 MΩ tip resistance) were fabricated from standard-wall borosilicate glass capillaries (B15023F, VitalSense) using a micropipette puller (P-97, Sutter Instrument). Whole-cell patch clamp experiments were performed using a MultiClamp 700B amplifier (Axon CNS). The intracellular solution was composed of 150 mM KCl, 2 mM MgCl_2_, 2 mM Na_2_ATP, 5 mM EGTA, and 10 mM HEPES (pH 7.2 adjusted with Tris). The extracellular solution was composed of 130 mM NaCl, 5 mM KCl, 2 mM CaCl_2_, 1 mM MgCl_2_, 10 mM sucrose, 20 mM glucose, and 10 mM HEPES (pH 7.4 adjusted with Tris).

### 2.6 Biocompatibility of the nanofibrous scaffolds

Cardiac progenitor cells were dissociated on day 6 post-differentiation and subsequently seeded onto nanofibrous membranes. A series of experiments were performed to evaluate the biocompatibility of PLCL/gelatin electrospun materials.

For cell adhesion analysis, 2 × 10^6 CPCs were cultured on the nanofibrous membranes for 48 h. Cellular morphology was assessed by staining filamentous actin (F-actin) following the manufacturer's protocol. Briefly, cells were fixed with 4 % paraformaldehyde (PFA), permeabilized with Triton X-100, and stained with F-actin Phalloidin Conjugates (Invitrogen) and 4′,6-diamidino-2-phenylindole (DAPI, Yeasen). Images were acquired using a confocal laser scanning microscope (TSC SP8, Leica, Wetzlar, Germany).

For cell viability and proliferation analyses, cells were seeded onto nanofibrous membranes or cell culture plates at a density of 7.5 × 10^5 CPCs/well. Cell proliferation was evaluated at 4 h, 1 day, 3 days, and 5 days using the Cell Counting Kit-8 (CCK-8, Dojindo) following the manufacturer's instructions. After incubation with CCK-8 reagent at 37 °C for 2 h, absorbance at 450 nm was measured using a microplate spectrophotometer (Multiskan MK3, Thermo Fisher Scientific, Waltham, Massachusetts, USA). Additionally, cells were incorporated with 10 μM EdU (Invitrogen) during different periods of cardiomyocyte differentiation. EdU labeling and nuclear staining were performed following the manufacturer's protocol to quantify cell proliferation. Confocal microscopy (TSC SP8, Leica, Wetzlar, Germany) was used to capture the images.

For cytotoxicity staining, CPCs and H9c2 cells were seeded onto PLCL/gelatin nanofibrous membranes. After 2 days of culture, the samples were collected, rinsed with PBS, and incubated with Calcein-AM and propidium iodide (PI) diluted at 1:1000 (Beyotime, C2015M) for 30 min. Fluorescence images were acquired using a confocal laser scanning microscope (TSC SP8, Leica, Wetzlar, Germany).

### 2.7 modRNA synthesis

Modified mRNA was synthesized via in vitro transcription using the MEGAscript™ T7 Transcription Kit (Invitrogen) from a linearized DNA template, with uridine fully substituted by N1-methylpseudouridine (m1Ψ). The open reading frame (ORF) sequences for human IGF1, enhanced green fluorescent protein (EGFP), and firefly luciferase were consistent with our previous studies [42,43]. Transcripts were purified using the MEGAclear™ Transcription Clean-Up Kit (Invitrogen), followed by enzymatic phosphorylation with Antarctic Phosphatase (New England Biolabs). After a re-purification step, RNA concentration was assessed using a Nanodrop 2000 (Thermo Scientific). All modRNAs were diluted to a concentration of 1 μg/μl and stored at −80 °C for further use.

### 2.8 modRNA transfection and quantification

Briefly, modRNA and MessengerMAX (Invitrogen) were separately incubated in Opti-MEM (Gibco) for 5 min at room temperature, followed by mixing for an additional 15 min to form liposome-modRNA complexes. The transfection mixture was diluted in Opti-MEM for cell transfection. After 4 h, the transfection medium was replaced with fresh culture medium. For each transfection, 2 μg of modRNA and 5 μL of MessengerMAX were used for approximately 1 × 10^6 cells.

Following modRNA transfection, EGFP fluorescence intensity was assessed using a fluorescence microscope (DMI3000 B, Leica, Wetzlar, Germany). To further evaluate the transfection efficiency of EGFP, cardiomyocytes were dissociated into single cells using the CardioEasy human cardiomyocyte digestion kit (Cellapy) and analyzed by flow cytometry (FACSCanto, BD Biosciences, Franklin Lakes, NJ, USA).

Enzyme-linked immunosorbent assay (ELISA, R&D Systems) was employed to quantify the secretion of IGF1 and to characterize the expression kinetics of modIGF1. Briefly, conditioned medium from cardiac patches containing 1 × 10^6 cells was collected at different time points (4h, 12h, 24h, 48h, 72h, 120h, and 1w post-transfection) and analyzed by ELISA according to the manufacturer's instructions. All experiments were performed in triplicate.

### 2.9 Flow cytometry analysis

Cells were dissociated into single cells and collected for intracellular staining. After fixation and permeabilization using Foxp3/Transcription Factor Staining Buffer Kit (Invitrogen) according to the manufacturer's instructions, cells were incubated with APC-conjugated antibodies against cTnT (130-106-689, Miltenyi) or subjected to indirect immunofluorescence. Specifically, cells were first stained with primary antibodies, including Islet1 (39.3F7, DSHB) and Tra-1-60 (MAB4360, Merck Millipore), followed by labeling with secondary antibodies, including Alexa Fluor 488-conjugated Donkey Anti-Mouse IgG H&L (ab150105, Abcam) and Alexa Fluor 488-conjugated Donkey Anti-Rabbit IgG H&L (ab150073, Abcam). The stained cells were analyzed using a BD FACSCanto™ Flow Cytometer, and data were processed with FlowJo_V10. All experiments were conducted in triplicate.

### 2.10 Cell proliferation and migration of human umbilical vein endothelial cells

For cell proliferation analysis, human umbilical vein endothelial cells (HUVECs) were seeded at a density of 1 × 10^5 cells per well in a 48-well plate and cultured with conditioned medium collected from modIGF1-transfected or luciferase modified mRNA (modLuciferase)-transfected cardiomyocytes within cardiac patches. Cell proliferation was evaluated using CCK-8 reagent at 1, 3, 5, and 7 days. At each time point, a 10 % (v/v) CCK-8 working solution was added to each well and incubated at 37 °C for 2 h. The supernatant was then transferred to a 96-well plate, and absorbance at 450 nm was measured using a microplate spectrophotometer (Multiskan MK3, Thermo Fisher Scientific, Waltham, MA, USA) under light-protected conditions.

For cell migration analysis, HUVECs were seeded in a 6-well plate. Upon reaching confluence, uniform scratches were created using a 200 μl pipette tip. Cells were then cultured in conditioned medium collected from modIGF1-transfected or modLuciferase-transfected cardiomyocytes within cardiac patches. Wound closure was monitored by capturing images at 0h, 24h, and 48h using a microscope (DMI3000 B, Leica, Wetzlar, Germany).

### 2.11 Construction of tissue engineering cardiac patch and histological staining

The electrospun nanofibrous membrane was cut into 15 mm diameter circular pieces and placed into a 24-well plate, pressed with a 10 mm inner diameter ring as previously described [36,44]. Cardiac progenitor cells (approximately 4 × 10^6 cells per well) were dissociated on day 6 post-differentiation and reseeded onto the nanofibrous membrane to construct the tissue-engineered cardiac patch. To improve cell viability during the initial 24 h, 5 μM Y27632 was added to the culture medium.

In vitro–constructed TECPs were embedded in optimal cutting temperature (OCT) compound and snap-frozen. Before fixation with 4 % PFA, the samples were sectioned into 7 μm slices using a cryostat. Standard hematoxylin and eosin (H&E; Solarbio) staining was performed according to the manufacturer's instructions. The stained sections were then dehydrated and mounted using neutral resin mounting medium, and images were acquired under a microscope.

### 2.12 Immunocytochemistry

Cells were dissociated and replated onto Matrigel-coated slides or electrospun membranes for immunocytochemistry. After collection, cells were fixed with 4 % PFA, permeabilized using 0.2 % Triton X-100, and blocked with 10 % goat serum for 2 h. Subsequently, they were incubated overnight at 4 °C with primary antibodies targeting Nanog (3369-1, Epitomics), Oct4 (2876-1, Epitomics), Sox2 (2876-1, Epitomics), Tra-1-60 (MAB4360, Merck Millipore), Islet1 (39.3F7, DSHB), cTnT (15513-1-AP, Proteintech), α-actinin (A7811, Sigma), Cx43 (ab11370, Abcam), and Mlc2v (ab79935, Abcam). On the following day, cells were incubated for 2 h at room temperature with Alexa Fluor 555- or Alexa Fluor 488-conjugated secondary antibodies (ab150106, Abcam; ab150073, Abcam), followed by nuclear counterstaining with DAPI (Yeasen) for 10 min. Fluorescent images were captured using a confocal laser scanning microscope (TSC SP8, Leica, Wetzlar, Germany).

### 2.13 RNA sequencing

Cardiac differentiation processes on nanofibrous membranes were depicted using RNA-sequencing among iPSC-CPCs (D6), early state iPSC-CMs (D10), and late state iPSC-CMs (D21). mRNA isolation and sequencing services were provided by BGI Genomics (Shenzhen, China). Briefly, the cDNA library was prepared according to the BGISEQ-500 protocol using Combinatorial Probe-Anchor Synthesis (cPAS) technology and assessed for quality using the Agilent Technologies 2100 Bioanalyzer. Sequencing data quality was controlled using Soapnuke, and gene expression levels were quantified using RSEM.

TECPs containing iPSC-CMs were transfected with either modIGF1 or modLuciferase on day 15 post-differentiation, followed by cell dissociation and collection 24 h post-transfection. mRNA isolation and sequencing services were provided by CloudSeq (Shanghai, China). Total RNA was extracted with Trizol (Invitrogen), and purified with the mRNA Purification Kit (GenSeq). Then, the purified mRNA was used for library construction with Directional RNA Library Prep Kit (GenSeq). Briefly, purified mRNA was fragmented (∼300 nt), and cDNA was synthesized using random hexamer primers. The resulting cDNA was end-repaired, dA-tailed, and ligated to adapters. After PCR amplification and purification, the libraries were sequenced with sequencer on the paired-end 150 bp mode. Paired-end reads were obtained and preprocessed using Fastp (v0.23.4) for quality control (Q30) and adapter trimming. High-quality reads were aligned to the reference genome with HISAT2 (v2.2.1), and mRNA expression profiles were generated with featureCount (v2.0.6).

### 2.14 Rat model of MI

All animal experiments were approved by the Laboratory Animal Welfare Ethics Committee of Shanghai Children's Medical Center (No. SCMC-LAWEC-2019-009). Male adult Sprague-Dawley rats, weighing approximately 200–250 g, were purchased from Shanghai Jiesijie Laboratory Animal Co Ltd. A rat model of MI was induced as previously described [35,42]. Rats were randomly assigned to the following five groups: a) Sham group: underwent the same procedures as MI group without coronary ligation; b) MI group: underwent MI without any treatment; c) MI-PA group: a cell-free nanofibrous membrane was sutured onto the heart surface after MI; d) MI-PA-iPS-CMs^modLuciferase^: a TECP containing modLuciferase-transfected iPSC-CMs was applied to the ischemic heart after MI; e) MI-PA-iPS-CMs^modIGF1^: a TECP containing modIGF1-transfected iPSC-CMs was placed onto the ischemic heart after MI. In detail, rats were placed on the operating table for endotracheal intubation and continuous anesthesia. After a left thoracotomy, myocardial infarction was induced by permanent ligation of the left anterior descending coronary artery using 6-0 sutures. TECPs were employed to cover the ischemic left ventricle using 12-0 sutures, and fibrin gel was applied to facilitate adhesion. The chest was then closed, and anesthesia was stopped to allow for animal recovery. To prevent immune rejection, all rats received tacrolimus (0.25 mg/kg/day) and methylprednisolone (5 mg/kg/day) every 12 h starting from the day before surgery until they were sacrificed. Both drugs were administered via oral gavage.

### 2.15 Triphenyltetrazolium chloride assay

The 2,3,5-Triphenyltetrazolium chloride (TTC, MP Biomedicals) assay was utilized as a metabolic marker to distinguish ischemic myocardial tissue from viable myocardium. A 2 % TTC solution (wt/v in PBS) was freshly prepared under light-protected conditions. At 24 h post-MI, rat hearts were harvested, rinsed with PBS, and frozen at −20 °C to achieve sufficient rigidity for sectioning. Each heart was transversely sliced into six 3 mm-thick sections and incubated in prewarmed TTC solution at 37 °C for 30 min in the dark. Following staining, the slices were washed with PBS and subsequently fixed in 4 % PFA. Finally, the heart sections were imaged against a black background, and the infarct area within the left ventricle (LV), including the septum, was quantified using ImageJ.

### 2.16 Echocardiography

Cardiac function analysis was performed as previously described [35,42]. In brief, transthoracic echocardiography was conducted at 1, 2, and 4 weeks post-MI. Echocardiographic recordings were obtained using the Vevo 2100 system equipped with a high-frequency MS-250 transducer (21 MHz) by a technician who was blinded to the experimental groups. Left ventricular internal diameters at end-systole (LVIDs) and end-diastole (LVIDd) were measured from the parasternal long-axis view using M-mode echocardiography. Key functional parameters, including left ventricular end-systolic volume (LVESV), left ventricular end-diastolic volume (LVEDV), left ventricular ejection fraction (LVEF) and fractional shortening (LVFS), were automatically calculated by the system's integrated software using the following equations: LVEF (%) = ((LVEDV-LVESV)/LVEDV) × 100 (%), LVFS (%) = ((LVIDd- LVIDs)/LVIDd) × 100 (%).

### 2.17 Heart morphology and histological evaluation

When rats were sacrificed at 1 week and 4 weeks post-MI, their hearts were collected and fixed in 4 % PFA overnight. The hearts were subsequently divided into specimens, dehydrated through an alcohol gradient, embedded in paraffin, and sectioned at a thickness of 6 μm.

After deparaffinization and rehydration, Masson trichrome staining (Solarbio) was performed according to the manufacture’s instructions. The percentage of the infarct size were analyzed with Image-Pro Plus 6.0.

For immunohistochemistry, paraffin-embedded heart sections were dewaxed and subjected to microwave antigen retrieval in citric acid buffer (pH 6.0, Yeasen). After permeabilization with 0.2 % Triton-X100 and blocking with 10 % goat serum, the sections were incubated overnight at 4 °C with primary antibodies: Ki67 (ab16667, Abcam), CD31 (ab182981, Abcam), Lamin A&C (ab108595, Abcam), αSMA (A5228, Sigma), and cTnT (15513-1-AP, Proteintech or ab8295, Abcam). Afterward, Alexa Fluor 555- or Alexa Fluor 488-conjugated secondary antibodies (ab150106, Abcam; ab150073, Abcam) were applied for 2 h at room temperature. Nuclei were stained with DAPI (Yeasen) for 10 min, and images were acquired using a confocal laser scanning microscope (TSC SP8, Leica, Wetzlar, Germany).

### 2.18 Statistical analysis

All data are presented as mean ± standard deviation (SD). Statistical analyses were performed using Student's t-tests, one-way ANOVAs, or two-way ANOVAs, depending on the data type and experimental design (analyzed with GraphPad). Statistical significance was defined as *p* < 0.05 for all tests. For all data in this study, “ns” indicates *p* > 0.05 (not significant), ∗ indicates *p* < 0.05, ∗∗ indicates *p* < 0.01, ∗∗∗ indicates *p* < 0.001, and ∗∗∗∗ indicates *p* < 0.0001.

## 3 Results

### 3.1 Fabrication and characteristics of the PLCL/gelatin nanofibrous membranes

**Fig. 2:**
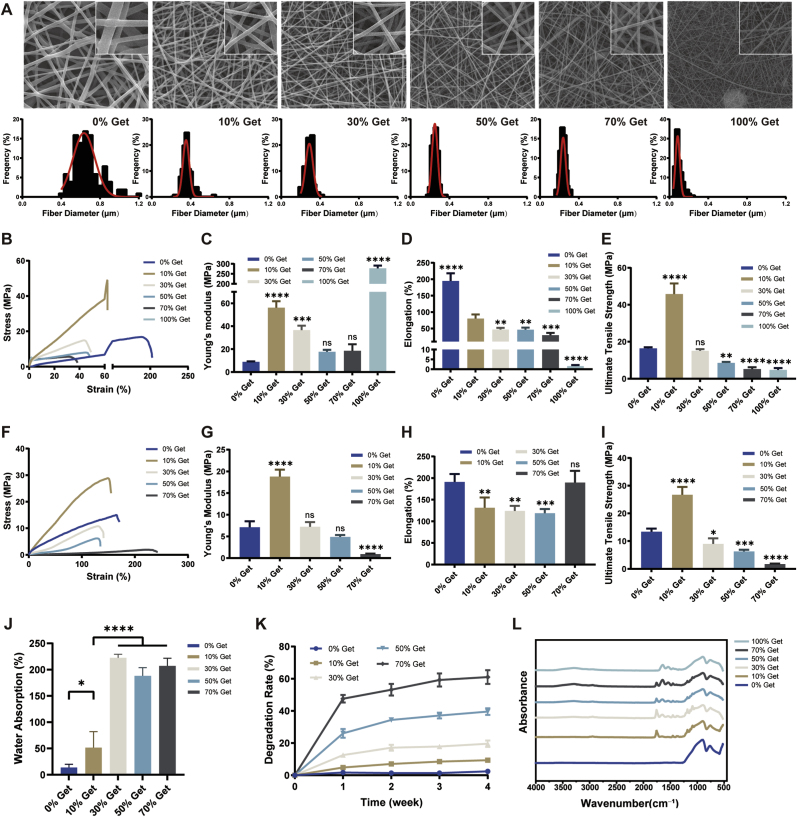
**Characteristics of PLCL/gelatin composite nanofibrous membranes.** (A) Representative SEM images and diameter distribution of the nanofibrous membranes in different groups. Scale bar = 5 μm. n = 100 per group. (B, F) Stress-strain curves of different nanofibrous membranes (B: dry, F: wet). n = 4. (C, G) Young's modulus of different nanofibrous membranes (C: dry, G: wet). n = 4. (D, H) Elongation at break of different nanofibrous membranes (D: dry, H: wet). n = 4. (E, I) Ultimate tensile strength of different nanofibrous membranes (E: dry, I: wet). n = 4. (J) Water absorption capability of different nanofiber membranes. n = 4. (K) Degradation rate of different PLCL/gelatin nanofibers at 1, 2, 3, and 4 weeks. n = 4. (L) FTIR spectra of different nanofiber membranes.

PLCL/gelatin composite nanofibrous scaffolds in varying proportions were synthesized using uniaxial blending electrospinning technology (Fig. S1A). Scanning electron microscopy was utilized to characterize the surface morphology and diameter distribution of these nanofibrous membranes. As illustrated in Fig. 2A, electrospun membranes in each group exhibited smooth and continuous nanofibers without noticeable rough beads. Quantitative analysis revealed that the fiber diameters for different PLCL/gelatin mass ratios of 100:0, 90:10, 70:30, 50:50, 30:70, and 0:100 were 0.67 μm ± 0.15 μm, 0.36 μm ± 0.05 μm, 0.28 μm ± 0.04 μm, 0.25 μm ± 0.03 μm, 0.24 μm ± 0.03 μm, and 0.10 μm ± 0.04 μm, respectively (Fig. 2A and S1B). It is evident that increasing the proportion of gelatin reduced the fiber diameter and improved the uniformity of the composite nanofibrous materials.

The representative stress-strain curves and tensile strength data for different PLCL/gelatin scaffolds were presented in Fig. 2B–I. Pure gelatin membranes broke instantly when stretched in the dry state and dissolved in the wet state, showing poor mechanical properties. For dry composite membranes, increasing gelatin content decreased the elongation at break. For wet composite membranes, elongation at break generally compromised with the higher gelatin proportions; however, the 70 % Get group exhibited comparable elongation to the 0 % Get group. Young's modulus and tensile strength initially increased from the 0 % Get group to the 10 % Get group but declined with further gelatin incorporation in both dry and wet nanofibrous membranes.

Water absorption serves as a key indicator for assessing the biocompatibility of biomaterials. As shown in Fig. 2J, PLCL/gelatin membranes containing 30, 50 and 70 wt% gelatin exhibited comparable water absorption, which was significantly higher than that of membranes containing 0 or 10 wt% gelatin. Furthermore, incorporation of gelatin significantly increased the degradation of PLCL/gelatin composite nanofibers, with a pronounced initial phase of mass loss during the first week and a gradual tapering of degradation kinetics in the subsequent weeks (Fig. 2K). These findings underscore the pivotal role of natural biomaterials in enhancing hydrophilicity, biodegradation, and biocompatibility.

To elucidate the chemical structures of PLCL/gelatin nanofibrous membranes, FTIR spectra of each group are presented in Fig. 2L. Characteristic absorption peaks of gelatin were observed at 3300 cm^−1^, 1645 cm^−1^ and 1545 cm^−1^, corresponding to the stretching of -OH and -NH, amide I and amide II, respectively. Additionally, blended nanofibers exhibited absorption bands at approximately 1760 cm^−1^ and 2945 cm^−1^, attributed to the vibrational modes of the -C=O and -CH_2_ groups in the PLCL molecular framework.

Considering the above analysis, while not altering the chemical structures, the optimal addition of gelatin could maintain the mechanical properties of PLCL to some extent and improve the biocompatibility of the nanofibrous membranes. PLCL/gelatin electrospun membranes containing 30 wt% gelatin was selected as the nanofibrous scaffolds for further experiments.

### 3.2 Adhesion and proliferation of iPSC-CPCs on PLCL/gelatin membranes

**Fig. 3:**
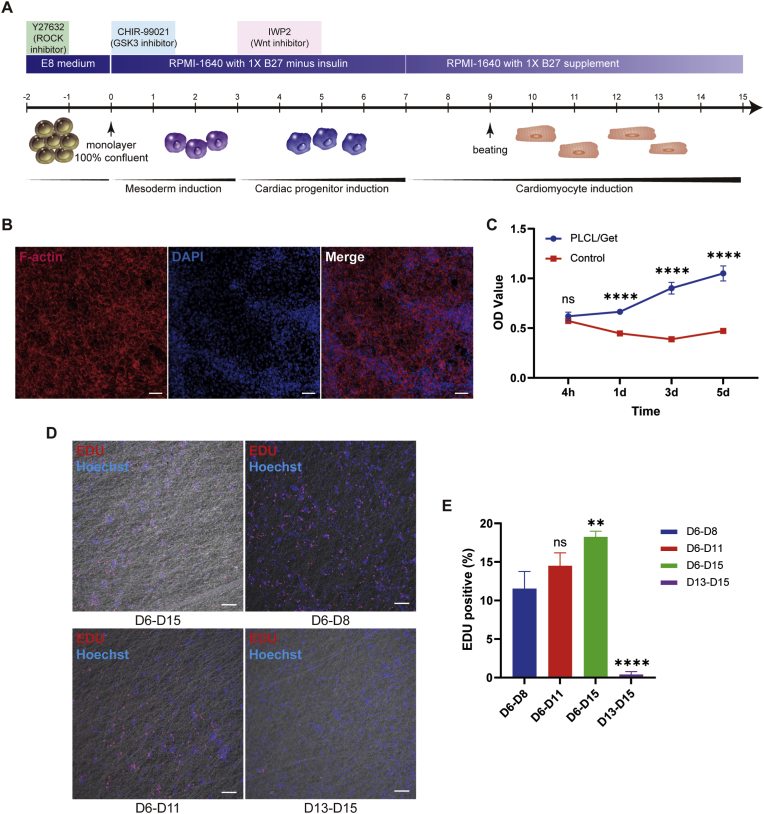
**Biocompatibility of PLCL/gelatin composite nanofibrous membranes.** (A) Schematic diagram of generating CPCs and CMs from hiPSCs. (B) F-actin staining of cells cultured on PLCL/gelatin nanofibrous membranes. Scale bar = 50 μm. (C) CCK-8 assay results at 4h, 1d, 3d, and 5d after seeding CPCs on PLCL/gelatin nanofibrous membranes and cell culture plates (Control). n = 4. (D-E) Representative EDU staining images (D) and quantitative analysis (E) of iPSC-CPCs cultured on PLCL/gelatin nanofibrous membranes at various different differentiation time points. Scale bar = 100 μm. n = 3.

Through modulating Wnt/β-catenin signaling pathway as previously described [[40], [41], [42]], it's feasible and reproducible to generate iPSC-CPCs and iPSC-CMs of high quality (Fig. 3A). Prior to cardiac differentiation, the pluripotent characteristics of hiPSCs were identified by labeling SOX2, NANOG, TRA-1-60 and OCT4 (Fig. S2A–D). The representative changes of cell morphology were observed during cardiac differentiation (Fig. S3A–B). On day 6 post-differentiation, iPSC-CPCs were identified using specific marker Islet1 (Isl1) (Fig. S3C), with the positive rate up to 81.83 % ± 5.48 % assessed by flow cytometry (Fig. S3D). By labelling with the myocardial marker cTnT and the pluripotent marker TRA-1-60, more than 80 % of the cells adopted the cardiomyocyte fate (Fig. S4A) while less than 1 % remained positive for TRA-1-60 (Fig. S4B) on day 15 post-differentiation, which suggested a minimal risk of tumor formation in the further in vivo applications. The clear sarcomere structures of iPSC-CMs were depicted by labelling myocardial markers cTnT and α-actinin (Fig. S4C). Transmission electron microscope (TEM) analysis further confirmed the presence of the characterized myofilament fibers, Z lines, mitochondria within iPSC-CMs (Fig. S4D). Ventricular-like action potential of iPSC-CMs were recorded using patch clamp technique (Fig. S4E), alongside the expression of the ventricular CM marker MLC2v (Fig. S4F), confirming that this differentiation method predominantly generates ventricular CMs. Additionally, the spontaneously robust beating of iPSC-CMs were observed under the microscope (Supplementary Movie 1).

The adhesion, survival and proliferation of iPSC-CPCs on the PLCL/gelatin composite nanofibrous membranes were further detected. Two days post-reseeding, we found that iPSC-CPCs could closely attach and fully stretch on the nanofibrous scaffolds (Fig. 3B). The proliferative rate of iPSC-CPCs on electrospun membranes was significantly higher than that on the cell culture plates (Fig. 3C). iPSC-CPCs were gradually differentiated into CMs and lost their proliferation potential during cell cultivation as assessed by EDU staining (Fig. 3D). Quantitative analysis showed that the EDU positive cells at D6-D8, D6-D11, D6-d15 and D13-d15 were 11.54 % ± 2.21 %, 14.50 % ± 1.67 %, 18.27 % ± 0.73 % and 0.41 % ± 0.36 %, respectively (Fig. 3E). To further assess the cytotoxicity of the scaffolds, Calcein-AM/PI staining demonstrated that 95.41 % ± 0.86 % of iPSC-CPCs remained viable on the PLCL/gelatin nanofibrous membranes. Given that CPCs exhibit a baseline level of spontaneous apoptosis during differentiation, we performed additional assays using H9c2 cells. The viability of H9c2 cells exceeded 99 %, confirming the excellent cytocompatibility of the composite nanofibers (Fig. S5A–C). These results provided evidence that PLCL/gelatin nanofibers containing 30 wt% gelatin could support a suitable microenvironment to maintain the attachment, growth, proliferation and cardiac differentiation of iPSC-CPCs.

### 3.3 Construction of TECPs in vitro

**Fig. 4:**
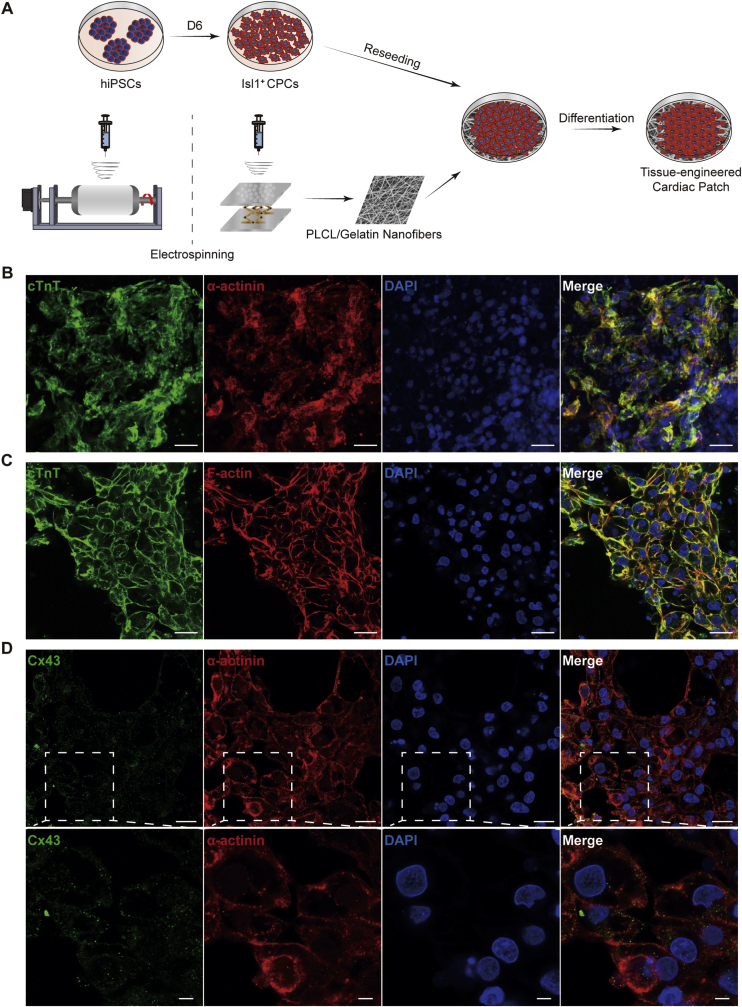
**Construction and immunofluorescence identification of tissue-engineered cardiac patch.** (A) Schematic illustration of in vitro TECP construction. (B–D) Immunofluorescence images of TECPs showing co-staining of cTnT and α-actinin (B, Scale bar = 25 μm), cTnT and F-actin (C, Scale bar = 25 μm), Cx43 and α-actinin (D, Scale bar = 25 μm. Scale bar for magnified images = 5 μm).

Electrospun PLCL/gelatin nanofibrous membranes and hiPSC-CPCs were prepared to formulate TECPs in vitro (Fig. 4A). To elucidate the spontaneous cardiac differentiation of iPSC-CPCs on PLCL/gelatin nanofibrous membranes, RNA sequencing was performed at different time points (D6, D10, and D21 post-differentiation). Comparative analysis of differentially expressed genes (DEGs) between D6 vs. D10 and D10 vs. D21 identified 103 persistently upregulated and 119 persistently downregulated DEGs, with representative DEGs visualized in heatmaps (Fig. S6A–B). Upregulated DEGs were primarily associated with muscle contraction, sarcomere organization, ventricular cardiac muscle tissue morphogenesis, voltage-gated calcium channel activity, and adrenergic signaling in cardiomyocytes (Fig. S6C–F). In contrast, downregulated DEGs were predominantly enriched in pathways related to tissue development, regulation of cell population proliferation, and cell cycle (Fig. S6G–J). The DEG analysis results aligned with the biological processes underlying cardiac differentiation. As differentiation progressed, myocardial-like tissues gradually formed and displayed spontaneous beating by day 15 post-differentiation (Supplementary Movie 2). Furthermore, H&E staining demonstrated that the in vitro–constructed TECPs developed a multilayered cellular architecture supported by the underlying nanofibrous scaffolds (Fig. S7). Additionally, immunofluorescence analysis confirmed the presence of sarcomere and cytoskeletal structure within TECPs (Fig. 4B and C). Connexin 43 (Cx43) expression was observed at the interfaces of adjacent CMs, indicating the establishment of gap junctions across TECPs (Fig. 4D). Together, these findings support the successful construction of TECPs using iPSC-CPCs and PLCL/gelatin scaffolds.

### 3.4 In vitro experiments of TECPs transfected with modified IGF1 mRNA

**Fig. 5:**
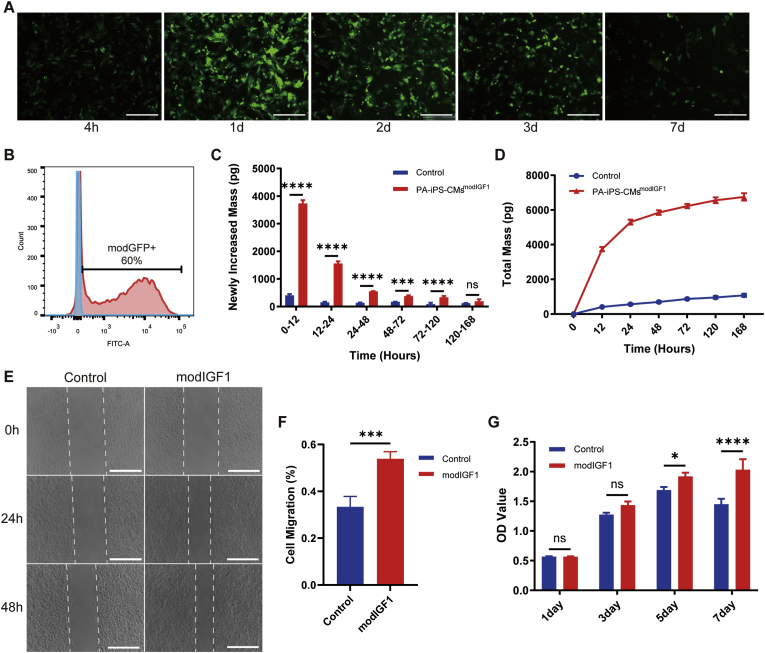
**Tissue-engineered cardiac patch transfected with modRNAs.** (A). Microscopic images showing GFP expression in hiPSC-CMs at different time points (4h, 1d, 2d, 3d, and 7d) following modGFP transfection. Scale bar = 200 μm, (B) Flow cytometry depicting the transfection efficiency of modGFP in hiPSC-CMs at 24 h post-transfection. (C–D) Quantification of newly synthesized IGF1 (C) and cumulative IGF1 (D) following modIGF1 transfection into TECPs at various time points. n = 3. (E) Cell migration assay of HUVECs at 0h, 24h, and 48h after treatment with conditioned medium from modIGF1-transfected and modLuciferase-transfected TECPs. Scale bar = 500 μm. (F) Quantitative analysis of HUVEC migration at 48h post-treatment with conditioned medium from modIGF1-transfected and modLuciferase-transfected TECPs. n = 4. (G) CCK8 assay assessing HUVEC proliferation at 1d, 3d, 5d, and 7d after treatment with modIGF1-transfected and modLuciferase-transfected TECPs. n = 3.

Our previous study demonstrated that N1-methyl-pseudouridine incorporated mRNAs, referring to modRNAs, could efficiently express proteins and enhance cell function [34,42]. The successful transfection of EGFP modified mRNA (modGFP) into hiPSC-CMs were confirmed by the detectable green fluorescence (Fig. 5A and Supplementary Movie 3). The expression kinetics followed a “pulse” pattern, peaking around 24 h after transfection and gradually decreasing over the first week. Flow cytometry analysis revealed a transfection efficiency of nearly 60 % for modGFP at 24 h post-transfection (Fig. 5B). To verify that TECPs could secrete the target protein IGF1 after modIGF1 transfection, cell culture supernatants were collected at certain time points and measured by ELISA assay. Quantitative analysis revealed that IGF1 production in the modIGF1 group was significantly higher than that in the control group during the first 5 days (Fig. 5C). Additionally, cumulative IGF1 protein levels in the modIGF1 group were consistently higher than those in the control group across all time points (Fig. 5D).

To confirm the functional activity of the secreted IGF1 protein, conditioned medium from modIGF1-transfected and modLuciferase-transfected TECPs was collected. Scratch wound healing and CCK8 assays indicated that modIGF1 transfection significantly enhanced the migration (Fig. 5E and F) and proliferation (Fig. 5G) of HUVECs, suggesting its potential to enhance angiogenesis.

**Fig. 6:**
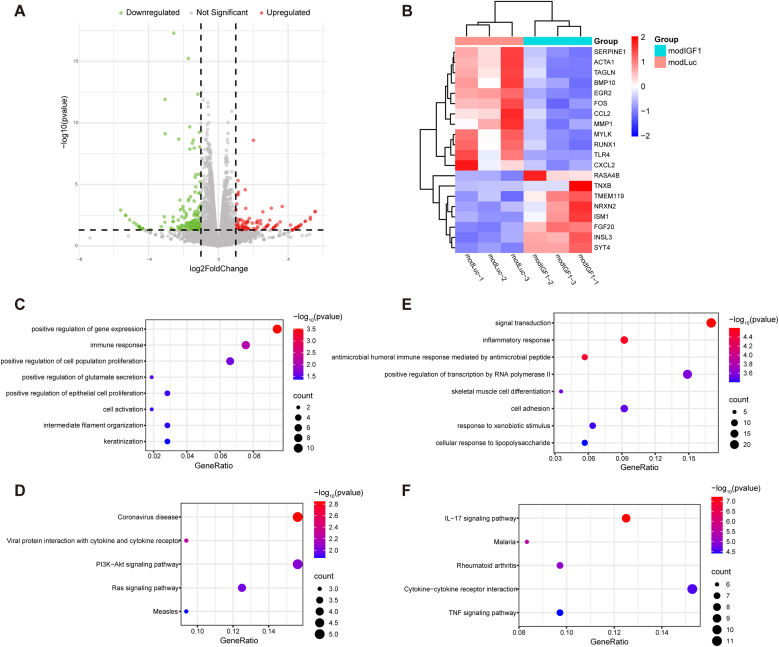
**RNA-sequencing results depicting differentially expressed genes after modIGF1 transfection.** (A) Volcano plot illustrating DEGs between the modIGF1-treated group and the modLuciferase-treated group. Red indicates up-regulated DEGs, green indicates down-regulated DEGs, and gray indicates non-significant DEGs. Note: The IGF1 gene itself has been excluded from the plot to enhance visualization of downstream effects. (B) Heatmap showing representative DEGs after modIGF1 transfection. Each rectangle represents a single gene, with blue indicating low expression and red indicating high expression. (C, D) GO biological process (C) and KEGG pathway (D) enrichment analyses of 106 upregulated DEGs following modIGF1 transfection. (E, F) GO biological process (E) and KEGG pathway (F) enrichment analyses of 141 downregulated DEGs following modIGF1 transfection.

RNA sequencing and subsequent analysis were performed to identify DEGs in TECPs following modIGF1 transfection. A total of 247 DEGs were identified between modIGF1-treated group and modLuciferase-treated group, including 106 upregulated genes and 141 downregulated genes (Fig. 6A and B). GO and KEGG enrichment analyses revealed that the upregulated genes were involved in processes such as cell proliferation, cell activation, PI3K-Akt and Ras signaling pathways (Fig. 6C and D), while the downregulated genes were associated with inflammation response, cell adhesion and skeletal muscle cell differentiation (Fig. 6E and F).

### 3.5 modIGF1-engineered TECPs improve cardiac function and morphology after MI

Rats were divided into 5 groups including Sham, MI, MI-PA, MI-PA-iPS-CMs^modLuciferase^ and MI-PA-iPS-CMs^modIGF1^, and sacrificed 4 weeks post-operation (Fig. S8A). Transmural myocardial infarctions were established by the ligation of left anterior descending coronary artery as previously described [35,42], after which modIGF1-engineered TECPs were transplanted to the infarct area (Fig. S8B and Supplementary Movie 4). TTC staining at 24h post-MI showed a clear break between the red viable myocardium and the white infarct region, confirming the success and consistency of the established MI models with the infarct size of (33.27 % ± 1.45 %) (Fig. S8C–D). The transplanted TECPs were visible on the surface of the infarct hearts without any post-operative adhesion in those groups with nanofibrous membranes (Fig. S9). Initial inspection found the evident scar formation in the infarct area of MI group and the presence of several small vessels on the surface of the TECP in MI-PA-iPS-CMs^modIGF1^ group.

**Fig. 7:**
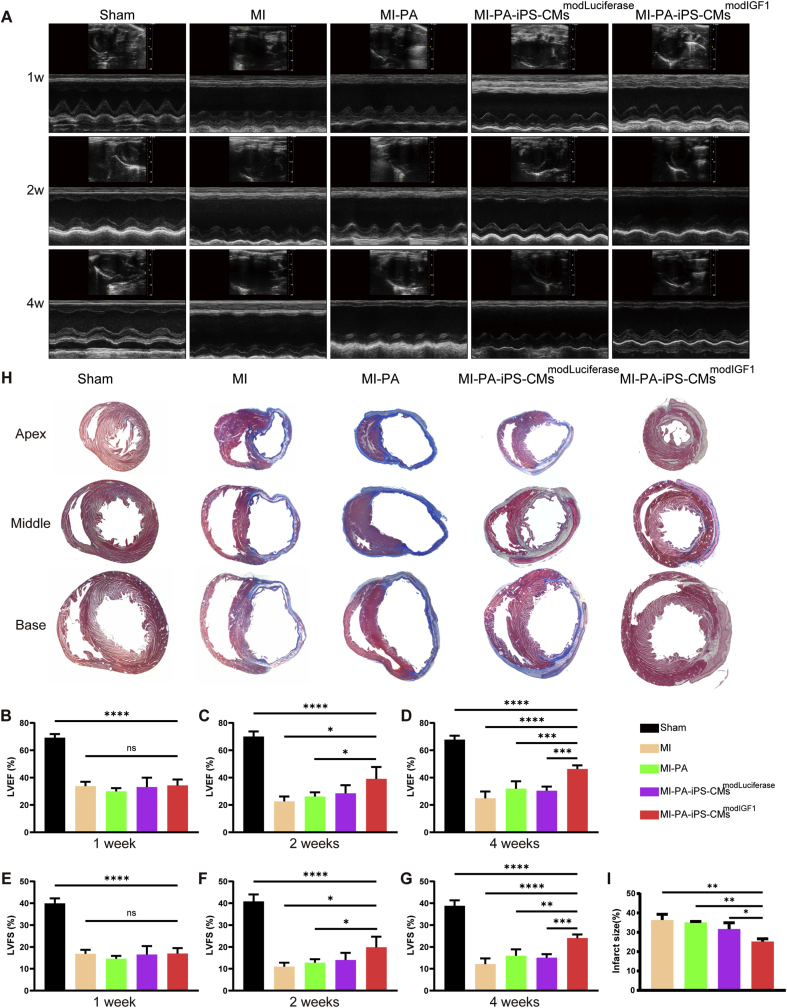
**Evaluation of heart function and histological morphology following myocardial infarction and cardiac patch transplantation.** (A) Representative M-mode echocardiogram recordings from each group at 1, 2, and 4 weeks after MI and cardiac patch transplantation. (B–D) LVEF of each group at 1 week (B), 2 weeks (C) and 4 weeks (D) after MI and cardiac patch transplantation. Sham, n = 4; MI, n = 3; MI-PA, n = 4; MI-PA-iPS-CMs^modLuciferase^, n = 5; MI-PA-iPS-CMs^modIGF1^, n = 4. (E–G) LVFS for each group at 1 week (E), 2 weeks (F) and 4 weeks (G) after MI and cardiac patch transplantation. Sham, n = 4; MI, n = 3; MI-PA, n = 4; MI-PA-iPS-CMs^modLuciferase^, n = 5; MI-PA-iPS-CMs^modIGF1^, n = 4. (H–I) Representative images (H) and quantitative analysis (I) of infarct size in rat hearts stained with Masson's trichrome at 4 weeks post-MI and TECP transplantation. MI, n = 3; MI-PA, n = 3; MI-PA-iPS-CMs^modLuciferase^, n = 4; MI-PA-iPS-CMs^modIGF1^, n = 4.

Echocardiographic examinations were performed at 1, 2, and 4 weeks after MI and TECP transplantation to assess cardiac function across the five experimental groups (Fig. 7A). Compared with Sham group, all MI groups demonstrated significant yet comparable impairments in cardiac function at 1 week post-surgery, as evidenced by reduced LVEF and LVFS (Fig. 7B and E). However, cardiac function of MI-PA-iPS-CMs^modIGF1^ group were significantly higher than that of MI and MI-PA groups at 2 weeks post-surgery (Fig. 7C and F). By 4 weeks post-surgery, modIGF1-engineered TECPs dramatically enhanced cardiac function (LVEF: 46.30 % ± 2.65 %; LVFS: 24.08 % ± 1.66 %), whereas treatment with modLuciferase-engineered TECPs (LVEF: 30.42 % ± 3.02 %; LVFS: 15.11 % ± 1.63 %) and non-cell cardiac patches (LVEF: 31.91 % ± 5.41 %; LVFS: 15.98 % ± 2.96 %) resulted in only modest functional improvement, compared with MI group (LVEF: 24.80 % ± 5.03 %; LVFS: 12.15 % ± 2.62 %) (Fig. 7D and G). These findings underscore the therapeutic efficacy of modIGF1-enhanced TECPs and highlight the potential of modRNA-based cardiac patch therapy.

Histological analyses were further conducted to assess the heart muscle morphology. Masson's trichrome staining demonstrated that the MI-PA-iPS-CMs^modIGF1^ treatment group exhibited a significantly reduced infarct size compared with other MI groups at 4 weeks post-operation (Fig. 7H and I). These findings suggest that modIGF1-engineered TECP treatment mitigates the severity of myocardial infarction and facilitates the recovery of cardiac function.

### 3.6 modIGF1-engineered TECP promotes angiogenesis, cell survival and proliferation

**Fig. 8:**
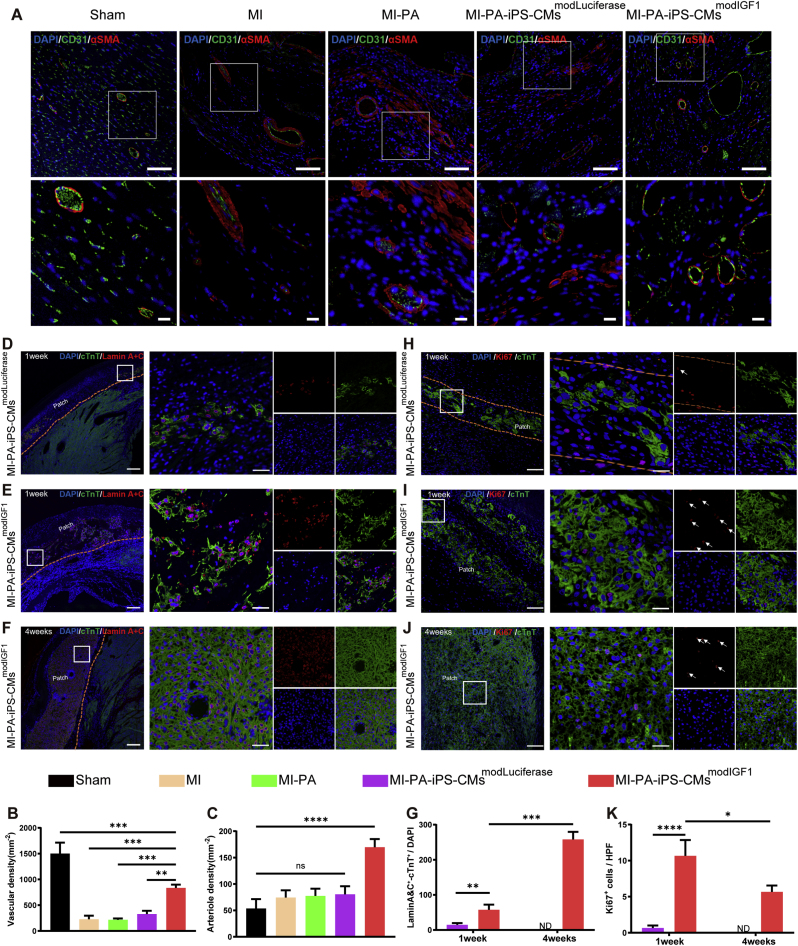
**modIGF1-treated TECPs enhance angiogenesis and promote transplanted cell survival and proliferation post-MI.** (A–C) Representative images (A) and quantitative analysis of vascular density (B) and arteriole density (C) within the infarct region, as determined by co-staining of CD31 and αSMA in rat hearts at 4 weeks after MI and TECP transplantation. Scale bar = 100 μm. Scale bar for magnified images = 15 μm. Sham, n = 3; MI, n = 3; MI-PA, n = 3; MI-PA-iPS-CMs^modLuciferase^, n = 4; MI-PA-iPS-CMs^modIGF1^, n = 4. (D–F) Representative images of Lamin A&C and cTnT immunostaining within modLuciferase-treated TECPs at 1 week (D) or modIGF1-treated TECPs at 1 week (E) and 4 weeks (F) post-surgery. Scale bar = 250 μm. Scale bar for magnified images = 25 μm. (G) Quantitative analysis of the viable CMs at 1 week and 4 weeks post-MI and TECP transplantation. n = 3 for 1 week. n = 4 for 4 weeks. (H–J) Representative images of Ki67 and cTnT immunostaining within modLuciferase-treated TECPs at 1 week (H) or modIGF1-treated TECPs at 1 week (I) and 4 weeks (J) post-surgery. The white arrows indicate the nuclear localization of Ki67. Scale bar = 75 μm. Scale bar for magnified images = 25 μm. (K) Quantitative analysis of Ki67 positive cells at 1 week and 4 weeks post-MI and TECP transplantation. n = 3 for 1 week. n = 4 for 4 weeks.

Neovascularization is critical for ensuring the prolonged retention and survival of transplanted cells in tissue-engineered cardiac patches. Co-staining of CD31 and SMA was performed to investigate the vascular network across five experimental groups at 4 weeks post-MI. The results revealed that MI-PA-iPS-CMs^modIGF1^ group exhibited a significant increase of capillaries in the infarct area compared with other treatment groups (Fig. 8A–C). Additionally, the co-localization of CD31 and α-SMA within the newly formed vessels in the MI-PA-iPS-CMs^modIGF1^ group suggests the development of mature vasculature.

The viability of CMs within TECPs was assessed through double staining for cTnT and nuclear Lamin A&C. Immunofluorescence analysis revealed that transplanted CMs were present in both MI-PA-iPS-CMs^modLuciferase^ and MI-PA-iPS-CMs^modIGF1^ groups at 1 week post-MI (Fig. 8D and E). However, viable CMs were only detectable in MI-PA-iPS-CMs^modIGF1^ group at 4 weeks post-MI (Fig. 8F). Quantitative assessments revealed that the survival rate of modIGF1-treated CMs was significantly higher than that of modLuciferase-treated CMs at 1 week post-MI, with a further increase observed at 4 weeks post-MI (Fig. 8G). Intriguingly, the low cell survival rate of modLuciferase-treated TECPs may explain the similar cardiac function results observed between MI-PA-iPS-CMs^modLuciferase^ and MI-PA groups. To further investigate the reason behind the increasing CMs, Ki67 staining analysis indicated that modIGF1 treatment significantly enhanced the proliferative potential of transplanted CMs compared with modLuciferase treatment at 1 week post-MI (Fig. 8H and I and 8K). However, the number of Ki67-positive CMs decreased by 4 weeks post-MI (Fig. 8J and K). These findings suggested that pre-treatment with modIGF1 promoted vascularization of the infarct region as well as enhanced cell survival and proliferation within the transplanted grafts, thereby potentially improving therapeutic outcomes.

## 4 Discussion

In this study, we applied modRNA technology for the first time to genetically modify tissue-engineered cardiac patches to allow for the controlled IGF1 secretion. Their beneficial effects on cell survival, proliferation, vascularization, and cardiac function were further evaluated in a rat model of MI. These findings offer a valuable foundation for advancing the application of modRNA technology in enhancing tissue-engineered cardiac patches.

In recent years, strategies employing living cells to restore cardiac structure and function have emerged as a promising avenue in regenerative medicine. However, their therapeutic efficacy has been hampered by significant hurdles, most notably the low viability of transplanted cells [45,46]. Previous studies sought to overcome the limitations by combining cell therapy approaches with biomaterials [3,47]. Electrospun nanofibrous biomaterials derived from synthetic and/or natural polymers hold significant promise for cardiac patch fabrication and have been extensively investigated for myocardial repair and regeneration [48,49]. In this study, nanofibrous membranes composed of PLCL and gelatin at varying ratios were fabricated via electrospinning technology. Characteristics analyses revealed that gelatin incorporation led to more uniform nanofibers, enhanced hydrophilicity and degradation rate, but compromised mechanical strength. Based on these findings, nanofibrous membranes containing 30 wt% gelatin was selected as the optimal scaffolds for cardiac patch construction. Human iPSC-CPCs provide a valuable cell source for cardiac tissue engineering, as they have been found to spontaneously differentiate into multiple cardiovascular lineages and retain the ability to proliferate, mature, and assemble into three-dimensional myocardial structures in vivo [50]. In parallel, numerous evidence indicates that myocardial constructs composed of heterogeneous cardiovascular populations exhibit superior structural organization, electromechanical coupling, vascular integration, and reparative efficacy compared with those generated from cardiomyocytes alone [[51], [52], [53], [54]]. Hence, iPSC-CPCs were employed as seeding cells to construct tissue-engineered cardiac patches in this study. The biocompatibility of PLCL/gelatin nanofibrous membranes were further assessed by seeding iPSC-CPCs onto the scaffolds, demonstrating robust support for cell attachment, growth, proliferation, and cardiac differentiation consistent with the upregulated signaling pathways including muscle contraction, sarcomere organization, ventricular cardiac muscle tissue morphogenesis, voltage-gated calcium channel activity, and the downregulated signaling pathways including tissue development, regulation of cell population proliferation, and cell cycle. Therefore, contractile TECPs were constructed by inoculating Isl1^+^ iPSC-CPCs on the nanofibrous membranes in vitro.

Another alternative strategy to improve engraftment efficiency has involved the use of genetic engineering techniques to modify donor cells, thereby enhancing their survival and reparative potential [55]. In the context of gene delivery approaches, mRNA stands out due to several practical and mechanistic advantages [56,57]. Its transient nature circumvents the risk of genomic integration and improves safety compared with viral vectors and plasmid DNA. In contrast to direct protein administration, mRNA supports prolonged endogenous protein expression. These properties render mRNA a flexible and effective platform for regenerative therapies. Our previous studies established a cell-based modRNA delivery system that enabled transient but efficient expression of target proteins and improved in vivo therapeutic efficacy in limb ischemia [34], myocardial ischemia [35,42], fat graft transplantation [58], defective bone repair [59] and random skin flap regeneration [60]. Additionally, we engineered vascular endothelial growth factor A (VEGFA) modified mRNA-transfected cellular electrospun membrane complexes (CEMCs) and emphasized their ability to improve cell survival and wound healing, supporting the feasibility and efficacy of integrating modRNA technology with tissue-engineered constructs [36]. Based on these findings, transient overexpression of therapeutic proteins via modRNA technology represents a promising strategy to enhance the implantation, functionality, and therapeutic effects in the field of cell therapies and tissue-engineered regenerative medicine. IGF1 has been extensively studied for its cardioprotective effects in ischemic myocardium. Studies have explored that short-term exogenous IGF1 treatment was sufficient to preserve cardiac function in animal models of ischemia-reperfusion injury [21,61]. In this study, in vitro experiments confirmed that TECPs transfected with modIGF1 could efficiently secrete IGF1 in a limited period, promoting endothelial cell migration and proliferation while upregulating signaling pathways related to cell proliferation, cell activation, and PI3K-Akt and Ras signaling, and downregulating signaling pathways such as inflammatory response and skeletal muscle differentiation. It was consistent with previous studies that IGF1 promoted cardiomyocyte proliferation via activation of PI3K/AKT signaling pathway [62,63]. Transplantation of modIGF1-enriched TECPs into ischemic myocardium revealed significant therapeutic benefits compared with other groups, including enhanced cell survival, increased cell proliferation, improved vascular formation within the infarct region, and mitigated cardiac dysfunction. These findings align with previous research supporting IGF1 as a cardioprotective factor [21,22,61,62], and provide evidence for tissue-engineered cardiac patches as an in vivo delivery platform for therapeutic cells enriched with key modified mRNAs to the infarct heart for the repair of MI.

Although graft attrition is frequently reported in cardiac cell transplantation studies, the increase in graft size observed at 4 weeks in the modIGF1 group is biologically plausible when considering both the developmental state of the transplanted cells and the effects of IGF1 mRNA modification. The in vitro cardiomyocyte differentiation protocols used in this study consistently generate a predominantly ventricular-like population of iPSC-CMs that retain an immature phenotype resembling embryonic CM [64,65]. Unlike adult cardiomyocytes, fetal or neonatal cardiomyocytes survive more readily after transplantation into injured myocardium [66]. Although more mature iPSC-CMs exhibit well-organized myofibrils, enhanced structural integration, and superior electrophysiological properties after transplantation [65,67], retaining partial immaturity—and thus proliferative potential—appears beneficial from a regenerative standpoint [68]. Consistently, our prior work has confirmed that the iPSC-CMs used here remain relatively immature and retain proliferative capacity in vivo, enabling measurable in situ graft expansion [42]. In addition, IGF1 mRNA modification further amplified these effects. Transcriptomic profiling revealed that IGF1 overexpression activated multiple pathways regulating cell proliferation, cellular activation, and PI3K–Akt signaling. These molecular signatures aligned with our immunostaining results, which showed substantially higher proportions of Ki67-positive cardiomyocytes within modIGF1 grafts at both 1 and 4 weeks. The presence of actively cycling cardiomyocytes provides direct evidence that proliferative expansion contributed to the observed increase in graft size. IGF1 is also well known to promote angiogenesis, thereby improving perfusion and enhancing the survival of transplanted cells. In our study, improved vascularization in the modIGF1 group likely created a more supportive microenvironment that further sustained graft survival and proliferation over time. Taken together, the immature proliferative phenotype of iPSC-CMs, along with the pro-survival and pro-angiogenic actions of IGF1, provides a coherent explanation for why grafts in the modIGF1 group expanded between 1 and 4 weeks. These findings underscore the distinct biological behavior of iPSC-CMs relative to their adult counterparts and highlight the need to consider the maturation state of transplanted cells when interpreting engraftment dynamics.

Compared with existing myocardial repair strategies, the modRNA-enhanced tissue-engineered cardiac patch offers a distinct and integrative approach. modRNA-mediated IGF1 expression in TECPs provides a robust, cell-driven source of IGF1 with precise spatiotemporal control, closely mimicking endogenous paracrine signaling. This approach overcomes the limitations of protein instability and allows the patch to act as a localized bioreactor for sustained therapeutic delivery. Moreover, modRNA-engineered cells benefit from enhanced survival, proliferation, and angiogenic support within the TECPs, whose effects cannot be fully recapitulated by protein release alone. Although the current study demonstrates the potential of modRNA-enhanced TECPs, the optimal modRNA dose, the maturation state of the iPSC-derived cells, and the structural design of the biomaterials remain to be further optimized to achieve maximal reparative outcomes. Overall, this study offers a reliable approach for enhancing the therapeutic potential of tissue-engineered cardiac patches and may further advance the application of modified mRNA technologies in regenerative cardiology.

However, this study has several limitations. First, we did not perform validation of the signaling pathways involved in the therapeutic effects of TECPs. Although our findings suggest potential molecular mechanisms, the absence of pathway validation limits the depth of mechanistic insights. Second, the cellular composition of TECPs is relatively complex. While most cells were cardiomyocytes, a small fraction of other cell types remained unidentified. Nevertheless, our observations demonstrated that a substantial number of cardiomyocytes survived within TECPs at 4 weeks post-transplantation, and the contribution of contaminating cells appeared limited.

## 5 Conclusion

In this study, surgically implantable tissue-engineered cardiac patches were constructed by integrating electrospun nanomaterials and iPSC-CPCs, and further engineered with modIGF1 to achieve controlled release of target proteins, thus significantly improving the therapeutic potential in the ischemic heart disease. The innovative incorporation of modRNA technology into tissue-engineered cardiac patch offers a novel perspective and makes a positive contribution to the field of cardiac tissue repair and regeneration.

## CRediT authorship contribution statement

**Bingqian Yan:** Writing – original draft, Visualization, Validation, Software, Project administration, Methodology, Investigation, Formal analysis, Data curation, Conceptualization. **Xuefeng Ai:** Writing – original draft, Visualization, Validation, Software, Project administration, Methodology, Investigation, Formal analysis, Data curation, Conceptualization. **Huijing Wang:** Writing – review & editing, Visualization, Investigation. **Yao Tan:** Writing – review & editing, Validation, Investigation. **Yiqi Gong:** Writing – review & editing, Visualization, Investigation. **Li Yang:** Writing – review & editing, Visualization, Investigation. **Ying Chen:** Writing – review & editing, Investigation. **Tingting Lu:** Writing – review & editing, Investigation. **Minglu Liu:** Writing – review & editing, Investigation. **Runjiao Luo:** Writing – review & editing, Investigation. **Kaixiang Li:** Writing – review & editing, Investigation. **Xin Tang:** Writing – review & editing, Investigation. **Wei Wang:** Writing – review & editing, Supervision, Resources, Funding acquisition. **Wei Fu:** Writing – review & editing, Supervision, Resources, Funding acquisition.

## Statement of significance

In this study, we report for the first time the application of novel mRNA technologies in combination with tissue-engineered cardiac patches (TECPs) to enhance heart regeneration. Nanofibrous membranes composed of polycaprolactone-co-l-lactide (PLCL) and gelatin were fabricated and screened out as scaffolds with favorable mechanical properties and biocompatibility to construct TECPs. We took an approach to autocrine insulin-like growth factor 1 (IGF1) protein in situ by transfecting IGF1 modified mRNA into TECPs just prior to cardiac transplantation in a rat myocardial infarction model. The transfection of TECPs with IGF1 mRNA ultimately led to greater survival rates of the transplanted cells, which may be attributed to the preserved proliferation capability of the induced pluripotent stem cell-derived cardiomyocytes (iPSC-CMs) and the promoted vascular network. The TECPs secreting IGF1 also aided in the tissue integrity of the heart muscle morphology and the cardiac function repair after myocardial infarction. This study may abridge mRNA technologies with tissue-engineered regenerative therapies in ischemic heart disease.

## Declaration of competing interest

The authors declare that they have no known competing financial interests or personal relationships that could have appeared to influence the work reported in this paper.

## Data Availability

Data will be made available on request.
