# Supplementary material for: Tissue-engineered cardiac patches enriched with IGF1 modified mRNA alleviate myocardial infarction by enhancing cell survival and angiogenesis
Source: Mater Today Bio. 2025 Dec 18;36:102686. doi: 10.1016/j.mtbio.2025.102686 (PMC12813182; doi:10.1016/j.mtbio.2025.102686)
Supplement: Multimedia component 1 [file mmc1.docx]

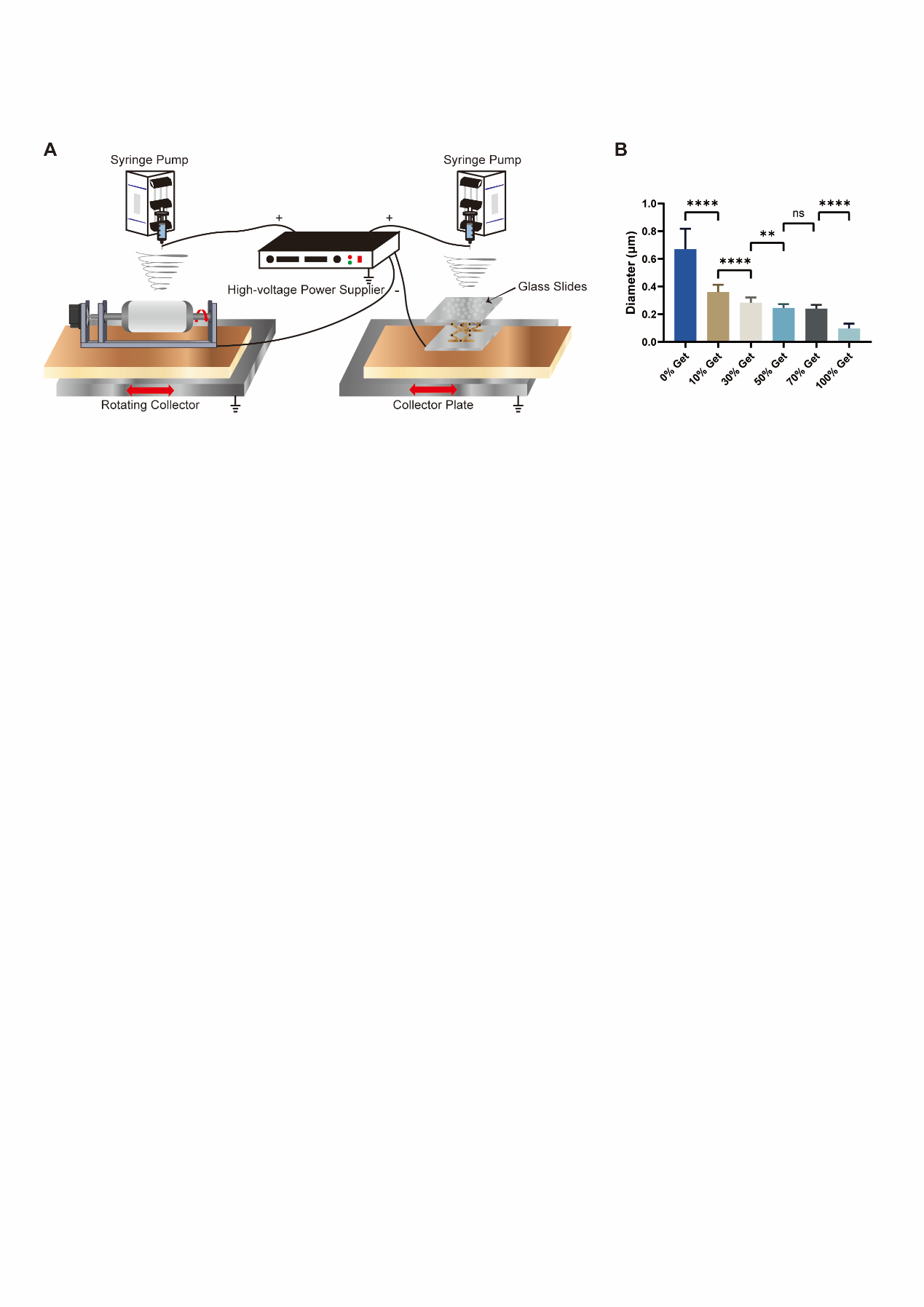


**Figure S1. Fabrication and diameter distribution of PLCL/ gelatin composite membranes.**

 (A) Schematic illustration of the fabrication process for PLCL/gelatin composite nanofibrous membranes. (B) Bar chart showing the diameter distribution of nanofibrous membranes in different groups. n = 100.


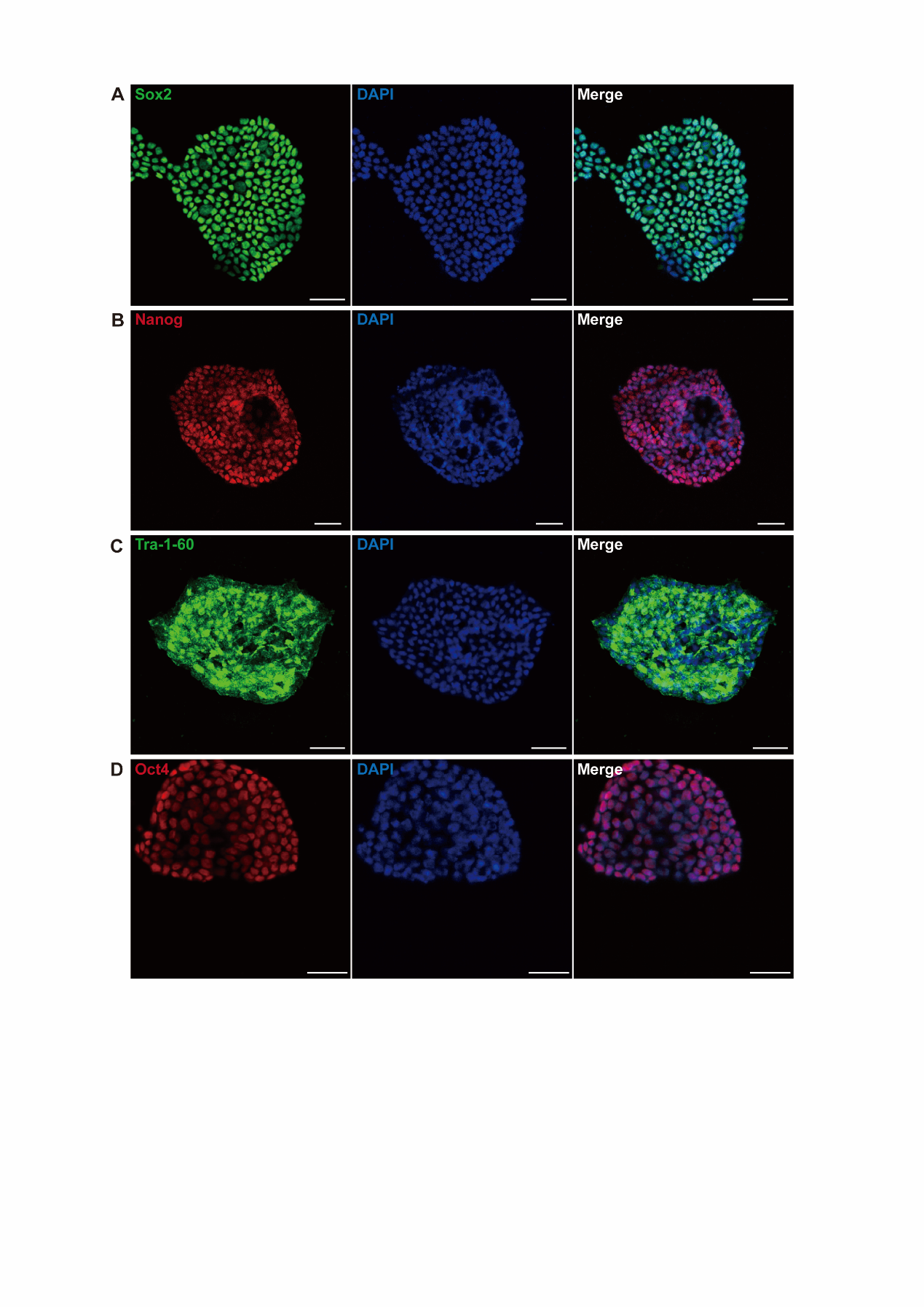


**Figure S2. Immunofluorescence staining of pluripotent markers in hiPSCs.** (A-D) Representative images of hiPSCs expressing Sox (A), Nanog (B), Tra-1-60 (C), Oct4 (D). Scale bar = 50 μm.


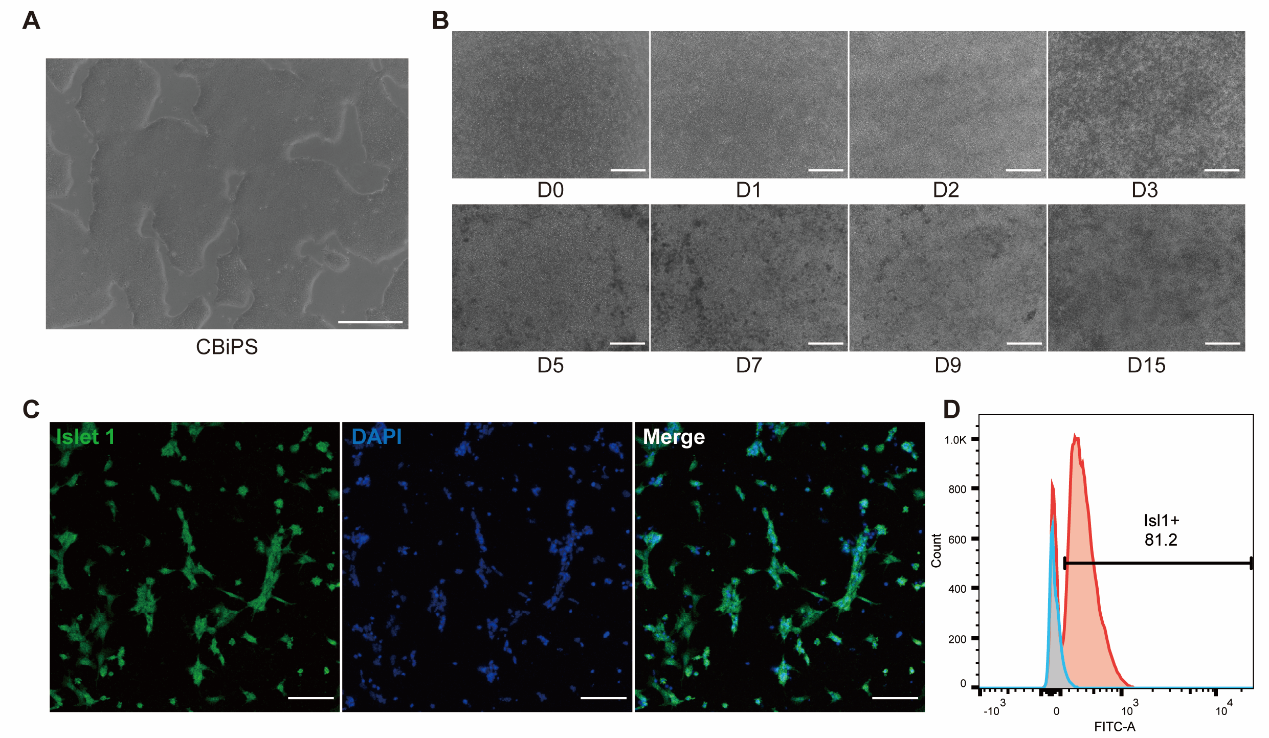


**Figure S3. Identification of hiPSC-CPCs.** (A) Representative microscopic image of hiPSCs. Scale bar = 500 μm. (B) Representative microscopic images showing morphological changes during the cardiac differentiation of iPSCs into CPCs and CMs. Scale bar = 500 μm. (C) Immunofluorescence staining of Islet1 in hiPSC-CPCs. Scale bar = 100 μm. (D) Flow cytometry analysis of Islet1 positive hiPSC-CPCs on day 6 post-differentiation.


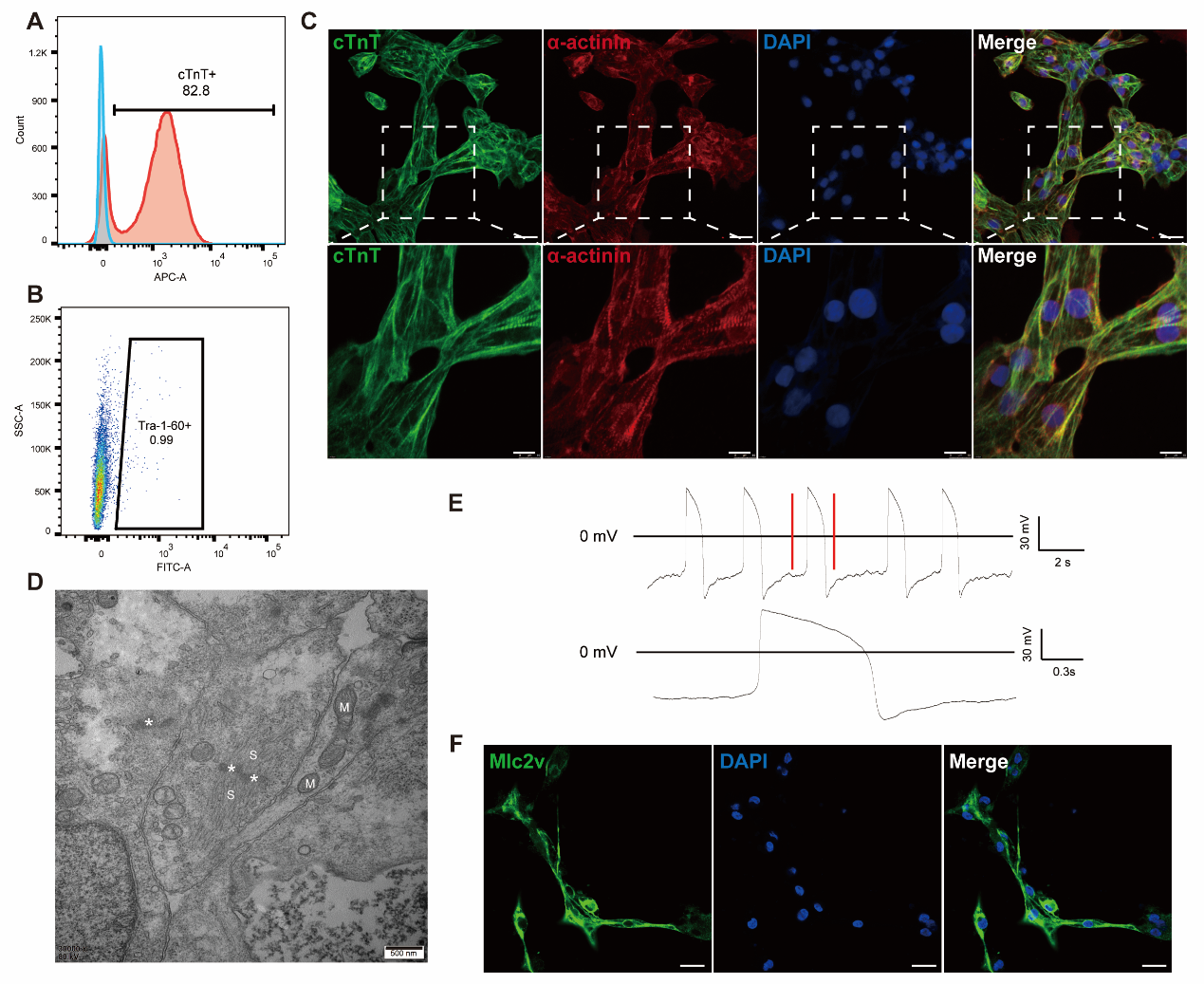


**Figure S4. Identification of hiPSC-CMs.** (A-B) Flow cytometry analyses of hiPSC-CMs expressing cTnT (A) and Tra-1-60 (B) on day 15 post-differentiation. (C) Immunofluorescence images of hiPSC-CMs expressing cTnT and α-actinin on day 15 post-differentiation. Scale bar = 25 μm. Scale bar for zoomed-in images = 10 μm. (D) Representative transmission electron microscopy （TEM） image revealing the structural details of hiPSC-CMs. Z line (white asterisk), mitochondrion (M), sarcomere (S). Scale bar = 500 nm. (E) Representative recordings of action potentials from a single hiPSC-CM using patch clamp. (F) Immunofluorescence images of hiPSC-CMs expressing Mlc2v on day 15 post-differentiation. Scale bar = 20 μm.


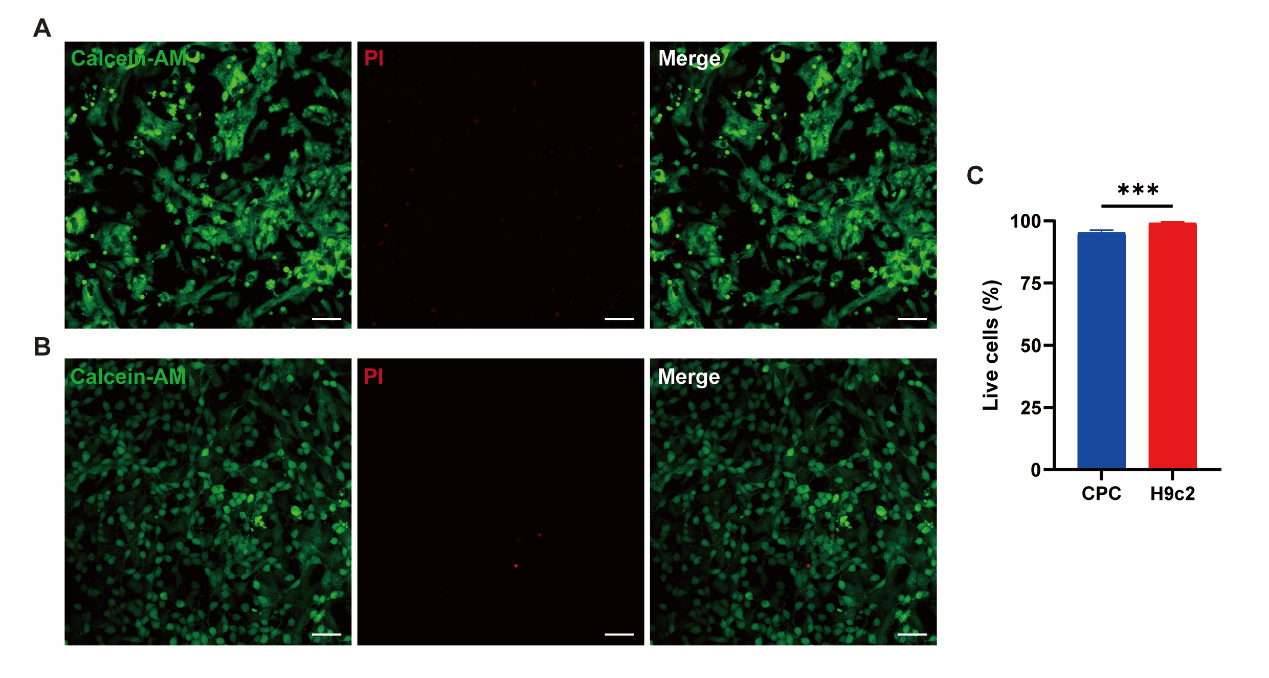


**Figure S5. Cytotoxicity of PLCL/gelatin nanofibrous membranes.** (A-B) Representative Calcein-AM/propidium iodide (PI) staining images of hiPSC-CPCs (A) and H9c2 cells (B) cultured on the PLCL/gelatin nanofibrous membranes for 48 hours. Live cells are labeled with Calcein-AM (green), while dead cells are labeled with PI (red). Scale bar = 50 μm. (C) Quantitative analysis of cell viability based on Calcein-AM/PI staining. n = 4.

**
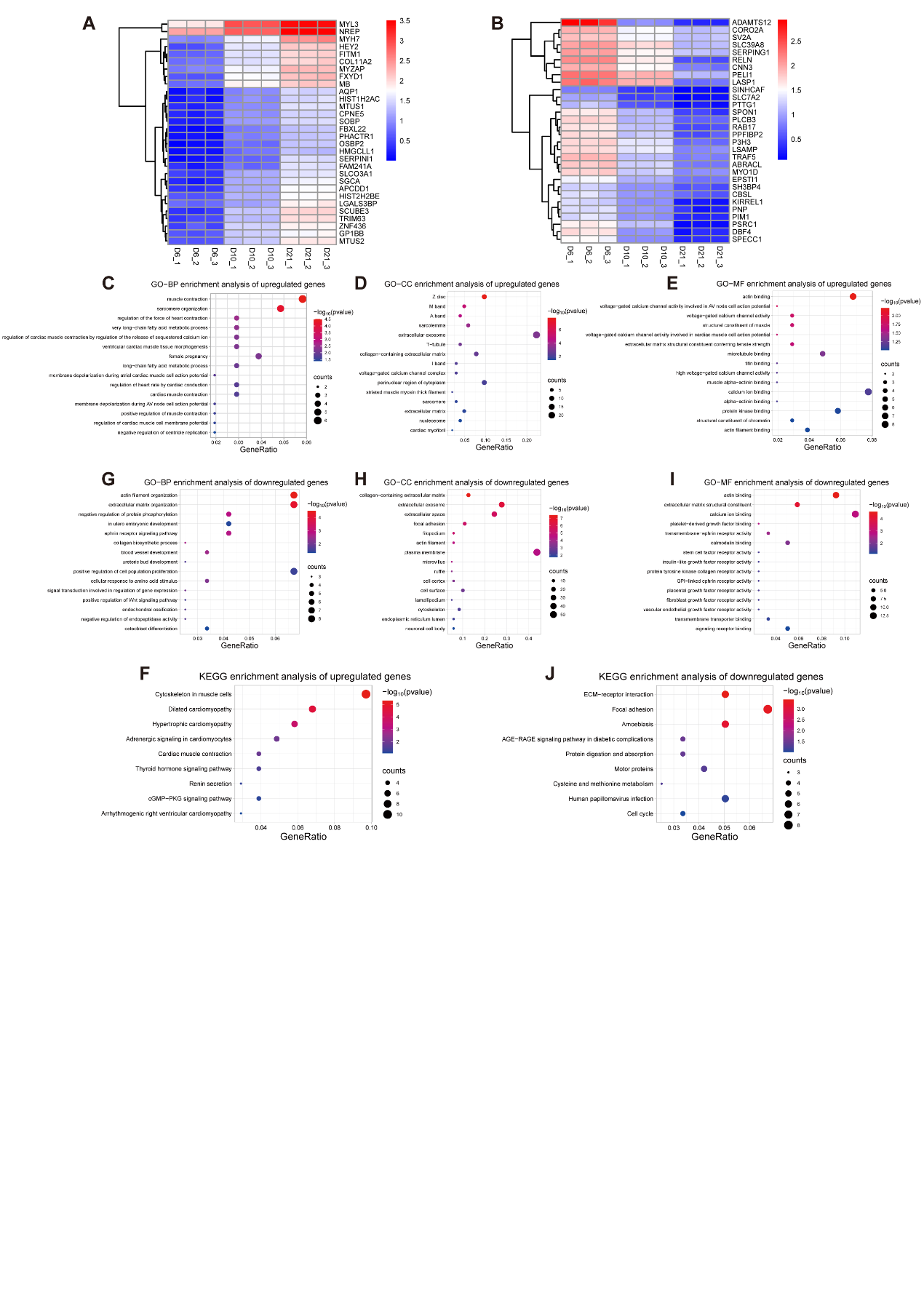
**

**Figure S6. Differential gene expression profiles during cardiac differentiation on PLCL/gelatin nanofibrous membranes.** (A-B) Heatmap displaying representative up-regulated (A) and down-regulated (B) differentially expressed genes (DEGs) at D6, D10, and D21 during cardiac differentiation. Each rectangle represents a single gene, with blue indicating low expression and red indicating high expression. (C-E) GO enrichment analysis of 103 persistently up-regulated DEGs across D6, D10, and D21 during cardiac differentiation, categorized into biological process (C), cellular component (D), and molecular function (E). (F) KEGG pathway enrichment analysis of the 103 persistently up-regulated DEGs during cardiac differentiation. (G-I) GO enrichment analysis of 119 persistently down-regulated DEGs across D6, D10, and D21 during cardiac differentiation, categorized into biological process (G), cellular component (H), and molecular function (I). (J) KEGG pathway enrichment analysis of the 119 persistently down-regulated DEGs during cardiac differentiation.


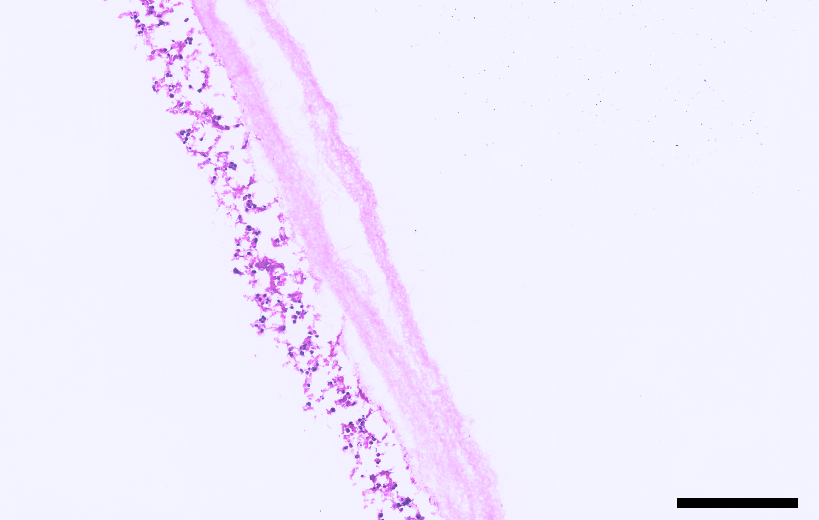


**Figure S7. Hematoxylin and eosin staining of in vitro–constructed tissue-engineered cardiac patches.** Representative H&E images showing the multilayered cellular architecture formed by hiPSC-CPCs seeded onto PLCL/gelatin nanofibrous scaffolds. Scale bar = 250 μm.


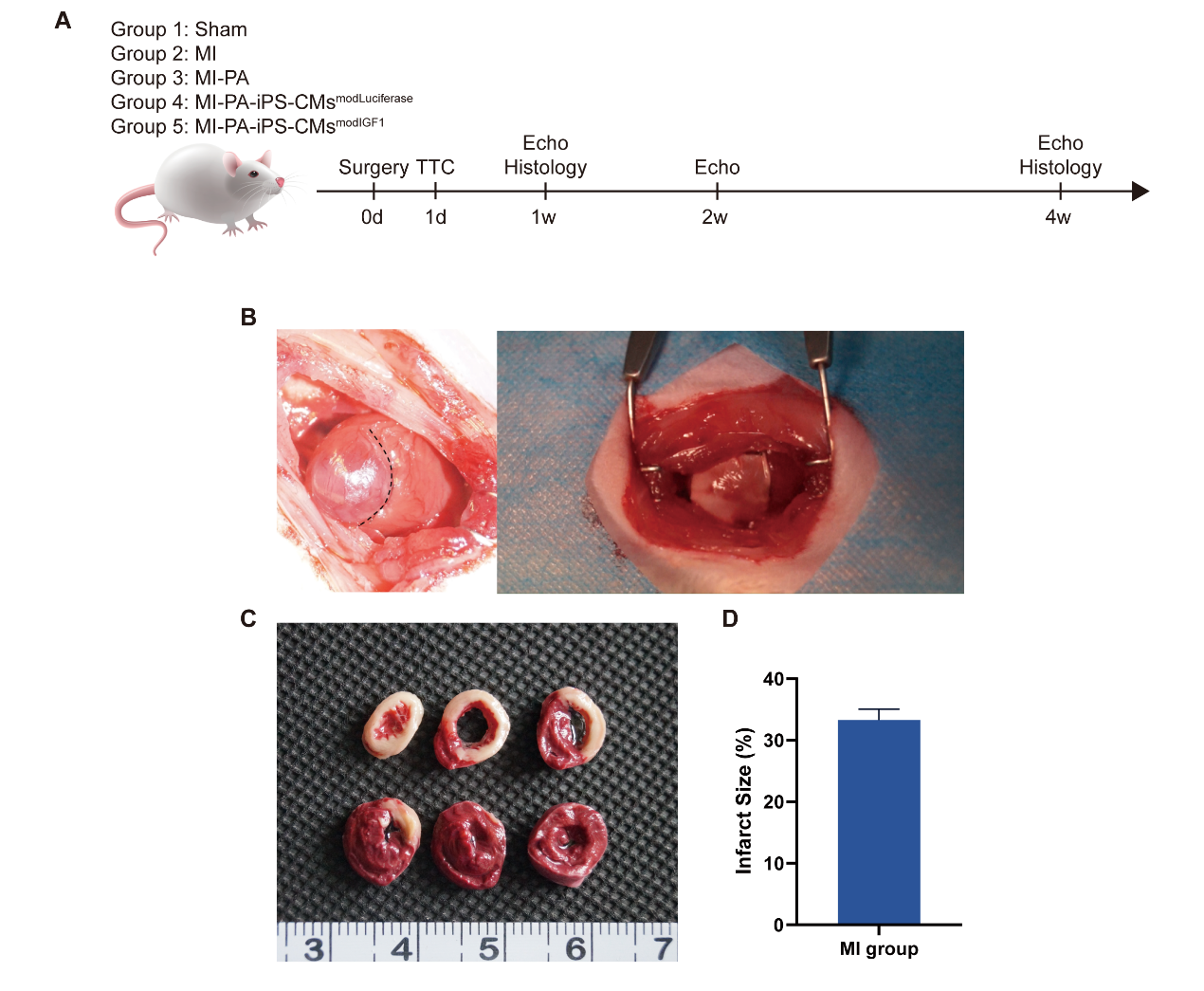


**Figure S8. Establishment of acute myocardial infarction in rat models.** (A) Schematic illustration of experimental grouping and procedures. (B) Representative images depicting myocardial infarction (MI) injury and the implantation of tissue-engineered cardiac patch. (C) Representative 2,3,5-triphenyltetrazolium chloride (TTC)-stained images demonstrating viable myocardium (red) and infarcted tissue (pale). (D) Quantitative analysis of the infarct size in MI models. n = 3.


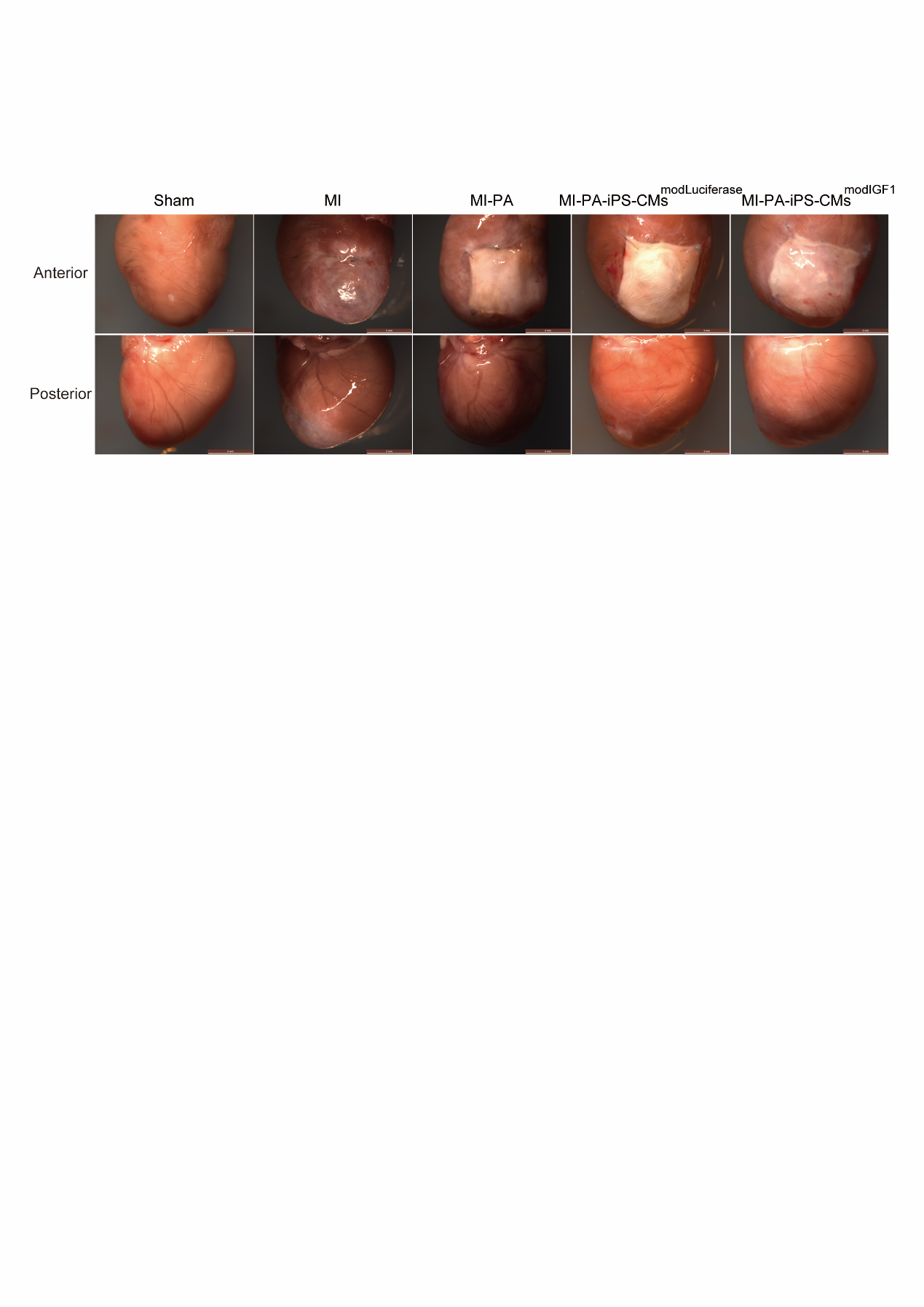


**Figure S9. Gross morphology of rat hearts at 4 weeks post-surgery.** Representative anterior and posterior views of rat hearts harvested 4 weeks after MI and TECP transplantation.

**Supplementary video 1.** Robust beating of iPSC-CMs

**Supplementary video 2.** Construction of tissue-engineered cardiac patch

**Supplementary video 3.** Transfection of modGFP into iPSC-CMs on day 15 post-differentiation

**Supplementary video 4.** Establishment of myocardial infarction and transplantation of tissue-engineered cardiac patch in a rat model
